# Physiologically based pharmacokinetic-quantitative systems toxicology and safety (PBPK-QSTS) modeling approach applied to predict the variability of amitriptyline pharmacokinetics and cardiac safety in populations and in individuals

**DOI:** 10.1007/s10928-018-9597-6

**Published:** 2018-06-25

**Authors:** Zofia Tylutki, Aleksander Mendyk, Sebastian Polak

**Affiliations:** 1 Unit of Pharmacoepidemiology and Pharmacoeconomics, Department of Social Pharmacy, Faculty of Pharmacy, Jagiellonian University Medical College, Medyczna 9 Str., 30-688 Krakow, Poland; 2 Department of Pharmaceutical Technology and Biopharmaceutics, Jagiellonian University Medical College, Medyczna 9 St, 30-688 Krakow, Poland; 3 Certara-Simcyp, Level 2-Acero, 1 Concourse Way, Sheffield, S1 2BJ UK

**Keywords:** Pharmacokinetic/pharmacodynamic (PK/PD) modeling, Pharmacokinetics, Physiologically based pharmacokinetic (PBPK) modeling, Toxicokinetics, Cardiac safety

## Abstract

The physiologically based pharmacokinetic (PBPK) models allow for predictive assessment of variability in population of interest. One of the future application of PBPK modeling is in the field of precision dosing and personalized medicine. The aim of the study was to develop PBPK model for amitriptyline given orally, predict the variability of cardiac concentrations of amitriptyline and its main metabolite—nortriptyline in populations as well as individuals, and simulate the influence of those xenobiotics in therapeutic and supratherapeutic concentrations on human electrophysiology. The cardiac effect with regard to QT and RR interval lengths was assessed. The Emax model to describe the relationship between amitriptyline concentration and heart rate (RR) length was proposed. The developed PBPK model was used to mimic 29 clinical trials and 19 cases of amitriptyline intoxication. Three clinical trials and 18 cases were simulated with the use of PBPK-QSTS approach, confirming lack of cardiotoxic effect of amitriptyline in therapeutic doses and the increase in heart rate along with potential for arrhythmia development in case of amitriptyline overdose. The results of our study support the validity and feasibility of the PBPK-QSTS modeling development for personalized medicine.

**Electronic supplementary material:**

The online version of this article (10.1007/s10928-018-9597-6) contains supplementary material, which is available to authorized users.

## Introduction

The physiologically based pharmacokinetic (PBPK) modeling approach has been used for various applications such as risk assessment for environmental health, academic research or drug development purposes [1, 2], in short, the safety and efficacy assessment. PBPK model parameters describing anatomy and physiology of the chosen species are compound-independent, which makes a model a universal framework for pharmacokinetics (PK) prediction in tissues of interest [3]. What is more, if properly parameterized, mechanistic PBPK models can predict inter-individual variability in drug’s PK profiles resulting from differences in human anatomy and physiology. A priori application of deterministic and/or stochastic approach in description of covariates (PBPK model parameters) affecting xenobiotics PK allows for predictive assessment of variability in a population of interest [1]. The next, future application of PBPK modeling that has already begun to be explored is in the field of precision dosing and personalized medicine. A profile of certain individual can be differentiated from a specific virtual population according to age, sex, and other specific physiological features [4, 5]. Such in silico models matching real patients, so called ‘virtual twins’, were also proposed by Polasek et al. [6] in order to predict individual olanzapine exposures and adjust the therapeutic dose. Zurlinden et al. [7] made use of that approach in the area of toxicokinetics, i.e., to predict paracetamol time-concentration profiles in humans under overdose condition, and to provide a method for ingested dose estimation. Patel et al. [8] simulated ‘virtual twins’, taking into account real patients’ physiology to mimic pharmacodynamics (PD), namely electrophysiological effect of citalopram taken, both in therapeutic and supratherapeutic doses.

Since, according to WHO, more than 300 million people suffer from depression [9], a large population is exposed to antidepressants. Although several new antidepressants were introduced, the old generation of tricyclic antidepressants (TCAs) are still in use despite being well-known for adverse cardiovascular effects [10]. Among TCAs, amitriptyline (AT) has been recognized to be most commonly associated with QT interval prolongation, arrhythmia, and the risk of sudden cardiac death [11]. The correlation between severity of the clinical manifestations of AT overdose and drug plasma levels is weak [12], so other individual factors should be taken into account in the attempts of prediction of drug adverse effects.

In this study we aimed to: (1) develop a PBPK model for AT administered orally, (2) simulate variability in PK of orally taken AT, and its main metabolite, nortriptyline (NT), with the use of PBPK model, (3) compare predictions versus clinically observed concentrations in differently characterized populations, (4) assess the ability of developed PBPK model extrapolation to simulate PK of overdosed real individuals, (5) estimate individual active cardiac concentrations of AT and NT, and their variability in the population, (6) simulate the effect of AT, and its main metabolite, NT, on human electrophysiology, both, observed clinically in populations and in overdosed patients (QSTS—Quantitative Systems Toxicology and Safety [13]) with the use of drug cardiac concentrations predicted in PBPK model.

## Methods

**Fig. 1:**
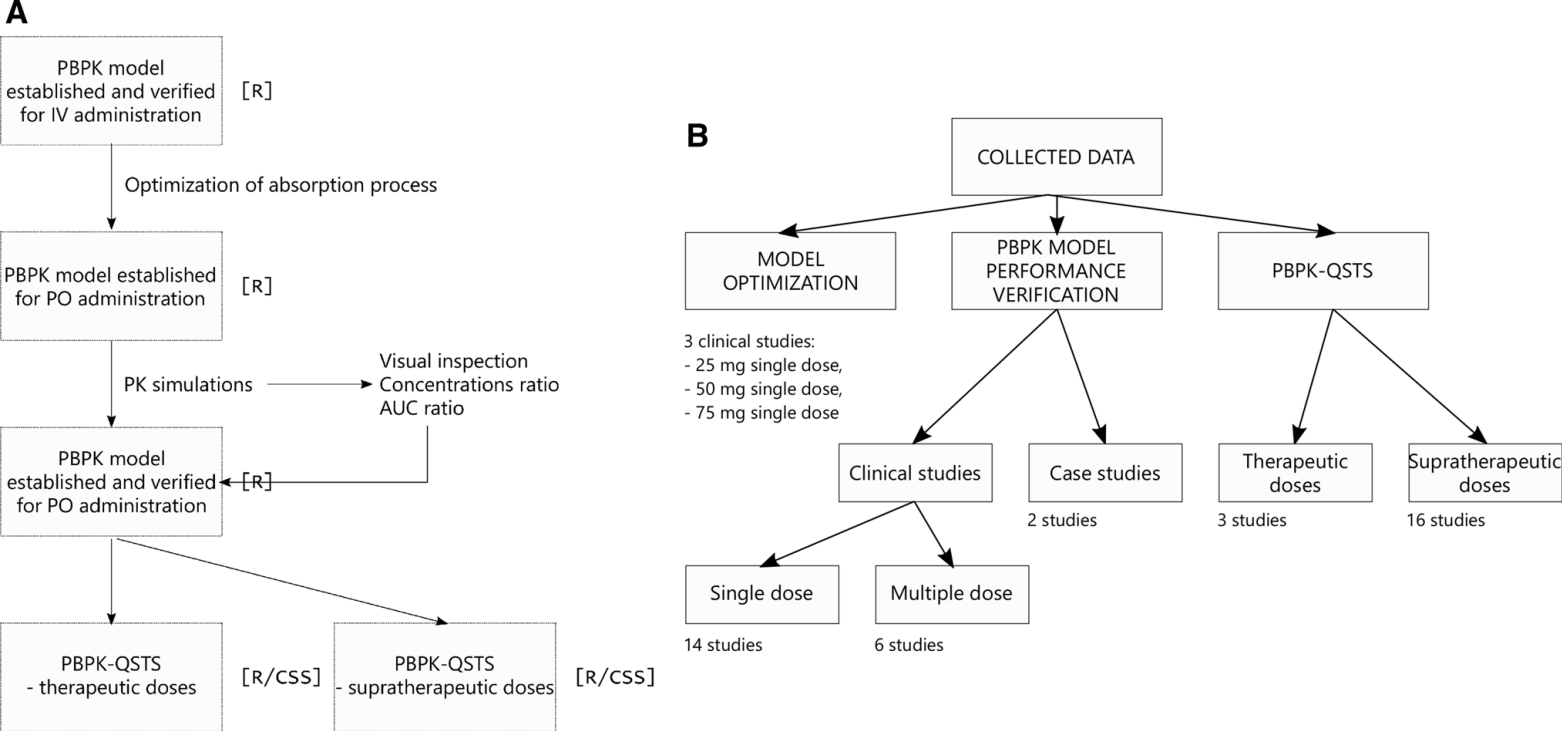
**a** The workflow of the study. The consecutive steps are listed in the blocks from top to bottom. The respective software was written in square brackets. **b** The exploitation of collected data for model optimization, PBPK model performance verification, and PBPK-QSTS modeling purposes

The workflow of the study and the exploitation of collected data is presented in Fig. 1.

### PBPK model structure

We used full-PBPK model developed for AT linked to minimal-PBPK model for the metabolite—NT [14] without changing any of the model parameters. The model had been built for intravenous AT administration and accounted for inter-individual variability. In order to implement the oral route of AT administration into the model, the drug absorption process was described with the equation of first order kinetics (Eq. 1):

1 $$\frac{{dDose_{F} }}{dt} = k_{a} \times Dose_{PO} \times F$$

where $\frac{{dDose_{F} }}{dt} \left[ {\frac{mg}{h}} \right]$ is the rate of taken orally AT entering the venous compartment, *k*_*a*_ [h^−1^] is the first order absorption rate, *Dose*_*PO*_ [mg] is the oral dose (po) of AT counted as a free base, and *F* is the AT bioavailability. Assuming:

2 $$F = (f_{a} \times F_{g} ) \times F_{h}$$

where f_a_ is the fraction of administered dose of AT absorbed to enterocytes, F_g_ is the fraction of AT escaping gut wall metabolism and entering portal vein, and F_h_ is a fraction of AT escaping liver metabolism during the first pass, the process of NT formation in the liver compartment in the first-pass metabolism was described as (Eq. 3):

3 $$\frac{{dA_{NT} }}{dt} = \frac{{MW_{NT} }}{{MW_{AT} }} \times (1 - \frac{F}{{(f_{a} \times F_{g} )}}) \times (f_{a} \times F_{g} ) \times Dose_{PO}$$

where $\frac{{dA_{NT} }}{dt} \left[ {\frac{mg}{h}} \right]$ is the rate of NT formation in the liver compartment in the first-pass metabolism, *MW*_*NT*_ [g/mol] *is* NT molecular weight, and *MW*_*AT*_ [g/mol] is AT molecular weight. AT absorption and NT formation were assumed to occur with mean lag time *t*_*lag*_ [h] with 30% CV. In case of multiple-dosage regimens patient-specific *t*_*lag*_ was assumed to be constant in time.

The values for the parameters describing AT absorption process were as follows:

*k*_*a*_—estimated in the optimization process, *Dose*_*PO*_—study-dependent, *F*—drawn from normal distribution of mean 0.459, standard deviation 0.093, truncated at 0.33 and 0.62 [15], (*f*_*a*_× *F*_*g*_*)*—drawn from log-normal distribution of mean 0.832 and coefficient of variation 0.131 [16], *MW*_*NT*_ equals 263.384 g/mol [17], *MW*_*AT*_ equals 277.4 g/mol [18], and *t*_*lag*_—estimated in the optimization process.

Two of the parameters of the absorption model, i.e., *k*_*a*_ and *t*_*lag*_ were fitted to the mean concentrations of AT and NT in plasma observed in three clinical studies after oral administration of AT [19–21]. These studies were chosen based on the criteria such as: representation of different doses of AT, mean plasma concentration reported both for AT and NT, inclusion of the Caucasian population (if not indicated directly, at least probable according to the authors affiliation). If two or more clinical studies characterized by the same dose of AT administered were fulfilling the criteria, the study with the PK reported for the longer period of time was chosen for fitting purposes.

The start values (intervals) set for both optimized parameters in the fitting process were set to 1 [0.1–2], and 1 [0–2] for *k*_*a*_ and *t*_*lag*_, respectively. The model cost was estimated as the root-mean-square error weighted by time (W-RMSE). Fitting was performed using the R statistical environment (version 3.4.1) with nloptr module used for global optimization and L-BFGS-B method of optim() procedure for local optimization. In the global optimization method controlled random search algorithm with local mutation (CRS2) was applied [22]. CRS2 is a global optimization method with constraints based on genetic algorithm coupled with random Nelder-Mead search strategy. After the CRS2 run with predefined number of iterations set to 1500 its solution was passed further to the L-BFGS-B method for refinement and final values of k_a_ and t_lag_ were obtained. The L-BFGS-B [23] method is a variation of classical quasi-Newton approach delivered by Broyden, Fletcher, Goldfarb and Shanno [24, 25]. This algorithm is capable of constrained optimization and therefore provides physically acceptable results of k_a_ and t_lag_ The number of L-BGFS-B iterations was set to 50 and relative tolerance stop criterion was set to 1e-20. For both approaches the internal optimization cost function was W-MSE (weighted mean squared error), transformed after the optimization into the W-RMSE for clarity of interpretation. The optimization runs were performed under Linux environment with R batch mode execution of R scripts.

### Pharmacodynamic models

The ten Tusscher ventricular cardiomyocyte cell model [26] implemented in the Cardiac Safety Simulator (CSS) v. 2.1 (Simcyp, Sheffield, UK, a Certara company) [27] was used to simulate pseudo-ECG traces. The CSS platform allowed for integration of the individual cardiac concentrations simulated in PBPK model, patient-specific information, and in vitro measured ion channels inhibition and consequent translation to in vivo human situation.

It is a known fact, that AT can modify the heart rate [28], therefore to describe the relationship between AT concentration and R–R interval length, an E_max_ model was established (Eq 4):

4 $$RR = \frac{{(RR_{0} - RR_{max} ) \times C^{n} }}{{EC_{50}^{n} + C^{n} }}$$

Were *RR* is the R–R interval length [ms], *RR*_*0*_ is the baseline R–R interval length [ms], *RR*_*max*_ is the maximum R–R interval length [ms], *EC*_*50*_ is the AT concentration that produces 50% of *RR*_*max*_, *C* is AT total plasma concentration [μM], and *n* is the sigmoidicity factor. The *E*_*max*_ model was fitted to literature-derived data [29–46] in R v. 3.4.0. with the use of simulated annealing “SANN” method of optimization from FME package [47].

### Model performance: therapeutic doses in populations

PBPK model performance verification was conducted by simulating clinical studies described in the literature where AT in therapeutic doses was administered orally. Simulations were run starting from the seed set to 1111. The results were compared with experimentally observed data, which were manually digitized from the published plots. The assessment of the model performance was based on:

1. visual inspection,
2. calculated ratios of the mean of predicted concentrations to mean of observed concentrations,
3. calculated ratios of predicted AUC to observed AUC.

Scientific literature resources were searched with the combinations of “amitriptyline”, “pharmacokinetics”, “clinical trial”, “QT” within PubMed/Medline and GoogleScholar. Twenty-four papers reporting PK of either AT alone or together with its metabolite, NT, after oral administration of AT in standard-release forms were identified. The populations described in the publications in question were either healthy or depressed with no other comorbidities. All of the identified studies were mimicked in modeling and simulation experiment. Three of them [19–21] served the purpose of parameters optimization. The other three [36, 48, 49], which contained data on time-matching QT measurement, entered the PK/PD modeling part of the study. The details of simulation scenarios are presented in Table 1 in Supplementary Material.

The simulated free AT and NT cardiac concentrations served as input values in CSS. The observed QTc values with time-matching QTc derived from simulated pseudo-ECG traces were compared. In QT length correction methods for heart rate we followed those described in clinical trials as close as possible.

### Model performance: clinical cases of AT intoxication

The established and verified PBPK model for oral administration of AT was used to predict individual toxicokinetic profiles (toxPK) of AT and NT in plasma and the heart tissue in case of AT overdose, and its impact on human electrophysiology. Scientific literature resources were searched with the combinations of “amitriptyline”, “overdose”, “intoxication”, “QT”, “TdP” within PubMed/Medline and GoogleScholar. Nineteen clinical cases of AT intoxication, in which there were no known other drugs altering cardiac electrophysiology taken, estimated dose and/or at least parent compound plasma concentration were reported, and time-matching QT (or QTc) measurement was available along with patient characteristics.

The found cases were divided into three groups with corresponding methodology:

1. Cases without ingested dose reported [35, 43]: the free cardiac AT and NT concentrations were estimated based on observed plasma AT and NT concentrations according to the (Eq. 5):
  5 $$C_{free} ,_{cardiac} = C_{total} ,_{plasma} \times Kp_{ht} \times fu_{ht}$$
  where C_free,cardiac_ is drug free cardiac concentration, Kp_ht_ is heart tissue to plasma partition coefficient, and fu_ht_ is drug unbound fraction in heart tissue. For AT, Kp_ht_ = 11.77, and fu_ht_ = 0.0012. For NT, Kp_ht_ = 35.63, fu_ht_ = 0.001. If plasma NT concentrations was unavailable, it was assumed to equal half of the observed AT plasma level.
2. Cases with both the estimated dose, and drug plasma concentrations reported [34]: ToxPK profiles were simulated in PBPK model and compared with observed plasma concentrations. The simulated free cardiac AT and NT concentrations were input into CSS for PD modeling. Since only the postdose time interval of ECG measurement was available, the cardiac concentrations corresponding to the simulated maximal plasma concentration of AT in that time interval were used.
3. Cases without drug plasma concentrations reported [12, 50–52]: The free cardiac concentrations of AT and NT were predicted in PBPK model based on the estimated toxic dose, and used as input in CSS.

All toxPK simulations were run ten times with the initial seed set to 1111. If the subject described in the clinical study claimed to take AT before the incident or suffer from depression, the toxic dose ingestion was simulated after the concentration reached steady-state. The data on individuals’ potassium, sodium, and calcium plasma levels, and RR interval length were taken into account if available in simulation of AT-triggered cardiac effect in CSS. In the case of no information on ions concentrations, they were assumed to be normal and the default settings in CSS were kept. In one case, i.e., the case reported by Paksu et al. [12], the RR was not available. Therefore in that case, the individual values of RR were predicted in E_max_ model based on simulated AT concentrations. The mean value of model derived RRs was input in CSS in Paksu study simulations.

The outputs of CSS were compared with the endpoints reported in case studies. It included: QT, QTcB, or torsade de pointes (TdP) arrhythmia event.

The details of simulation scenarios are presented in Table 1 in Supplementary Material.

## Results

### Estimates of the parameters

The W-RMSE estimated for initial values of optimized parameters was 10.01 and decreased to 9.24 after running the model-fitting algorithms. All computations were run on a multiserver, multicore grid, working under the control of job control system. Despite that, there was no significant reduction of fitting, thus we conclude that the model is stable and its parameters represent the closest possible estimates of the final values. The estimates were as follows: *k*_*a*_= 0.24 [h^−1^] and *t*_*lag*_= 1.33 [h].

### E_max_ model

**Fig. 2:**
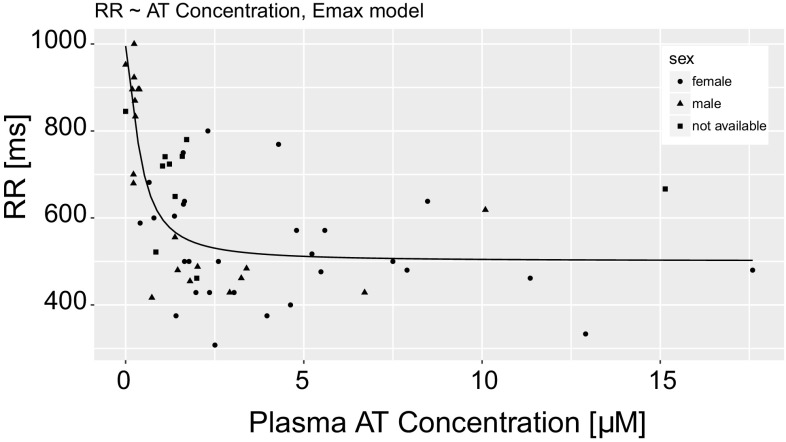
The E_max_ model total plasma AT concentration [μM] − RR interval length [ms] curve along with the values clinically observed (dots)

The estimates of E_max_ model parameters were as follows:

*RR*_*0*_ = 995.3 [ms], *RR*_*max*_ = 500.8 [ms], *EC*_*50*_ = 0.4 [μM], and *n *= 1.5. The RMSE of established E_max_ model equaled 120.98. The RR interval length versus AT plasma concentration simulated in E_max_ model is depicted in Fig. 2 along with the values clinically observed.

### Model performance: therapeutic doses in populations

**Fig. 3:**
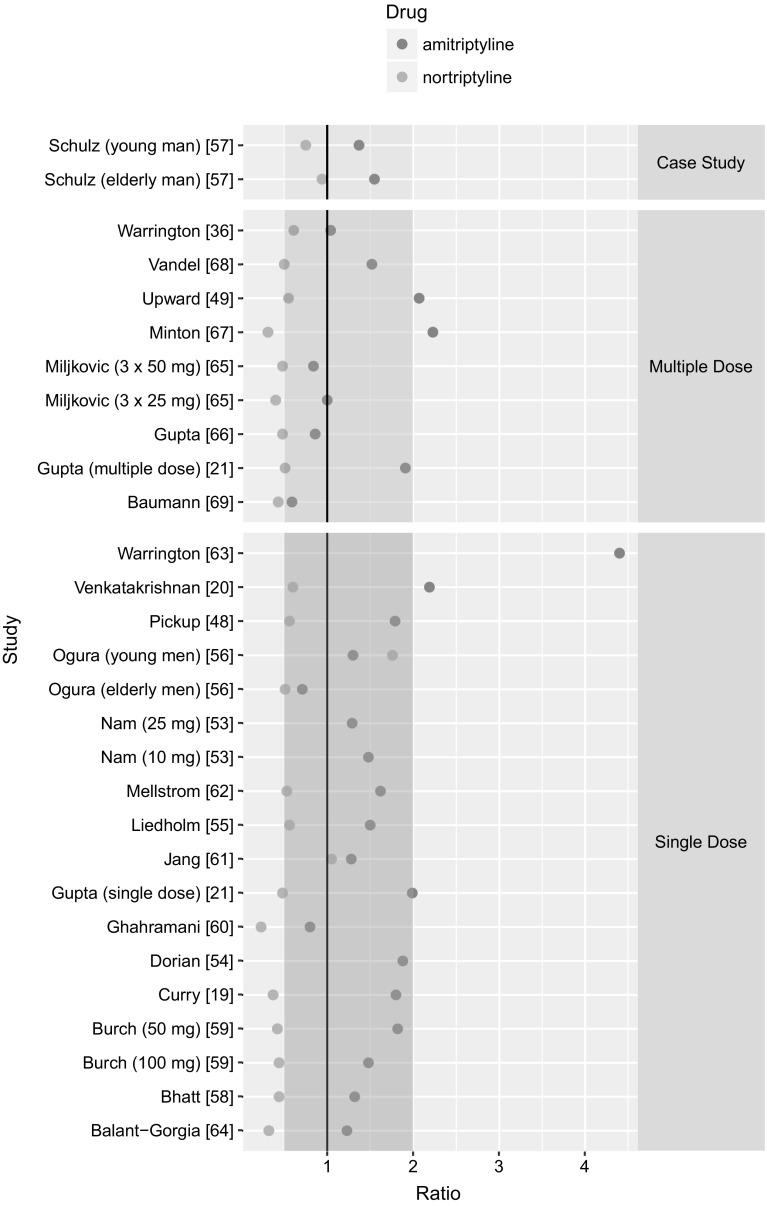
The ratio of predicted mean concentrations to observed mean concentrations of AT (red dots) and NT (blue dots). Two-fold margin is marked in pink (Color figure online)

With the use of PBPK model, 29 trials in which AT was administered orally either as a single dose (20 studies) or in multiple dosage schedules (9 studies), were mimicked. Regarding single AT dose administration, the PK after following doses was simulated: 10 mg [53], 25 mg [19, 53–56], 40 mg [57], 50 mg [20, 58–63], 75 mg [21, 48, 64], 80 mg [57], and 100 mg [59]. The simulated PK profiles along with AT and NT (if available) concentrations observed in the clinic are presented in Fig. 1 A-AC in Supplementary Material. The predicted mean concentrations of AT were within two-fold of their respective observed means for 18 (out of 20) studies (Fig. 3).

**Fig. 4:**
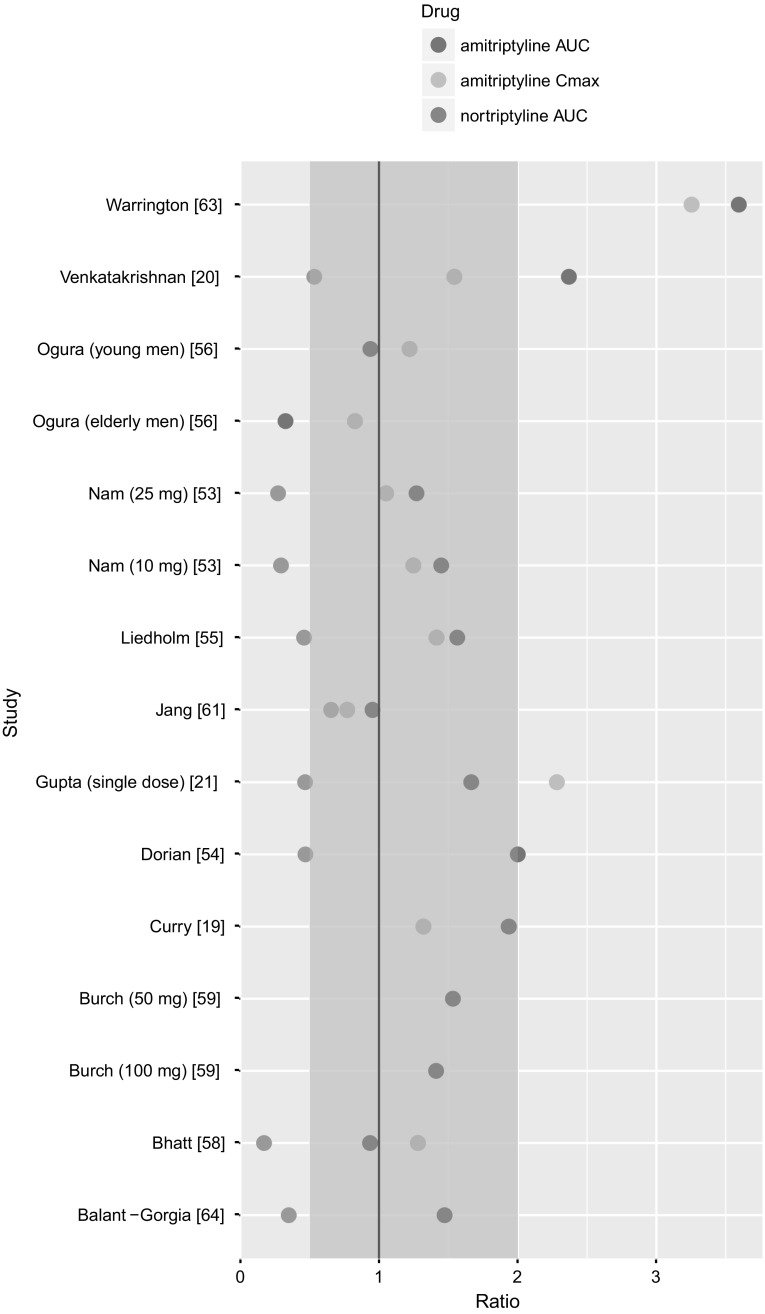
The ratio of predicted to observed dose metrics: AUC for time-concentration profile of AT (red dots), AUC for time-concentration profile of NT (blue dots), C_max_ for AT (green dots). Two-fold margin is marked in pink (Color figure online)

The simulated AUC of AT were within two-fold of their respective AUC derived from clinically observed data for 12 (out of 15 for which the AUC value was reported) studies (Fig. 4).

The simulated C_max_ for AT were within two-fold of their respective clinically observed C_max_ for 9 (out of 11 for which the C_max_ value was reported) (Fig. 4). The predicted mean concentrations of NT were within two-fold of their respective observed means for 8 (out of 15) studies (Fig. 3). The simulated AUC of NT were within two-fold of their respective AUC derived from clinically observed data for 5 (out of 9 for which the AUC value was reported) studies (Fig. 4).

Regarding multiple AT dose administration, the PK after the following dosage schemes was simulated: 25 mg q.d. [21], 25 mg t.i.d. [65], 50 mg t.i.d. [65], 75 mg q.d. [66, 67], 125 mg q.d. [68], AT in ascending doses, i.e., 100 mg–150 mg–200 mg q.d. [49], 75 mg–150 mg [36, 69]. The predicted mean concentrations of AT were within two-fold of their respective observed means for 7 (out of 9) studies. The predicted mean concentrations of NT were within two-fold of their respective observed means for 6 (out of 9) studies (Fig. 3).

Free AT and NT cardiac concentrations simulated under the scenarios of three clinical trials [36, 48, 49] were further used as input values in CSS to mimic the electrophysiological effect of administered drug measured in those clinical trials.

**Fig. 5:**
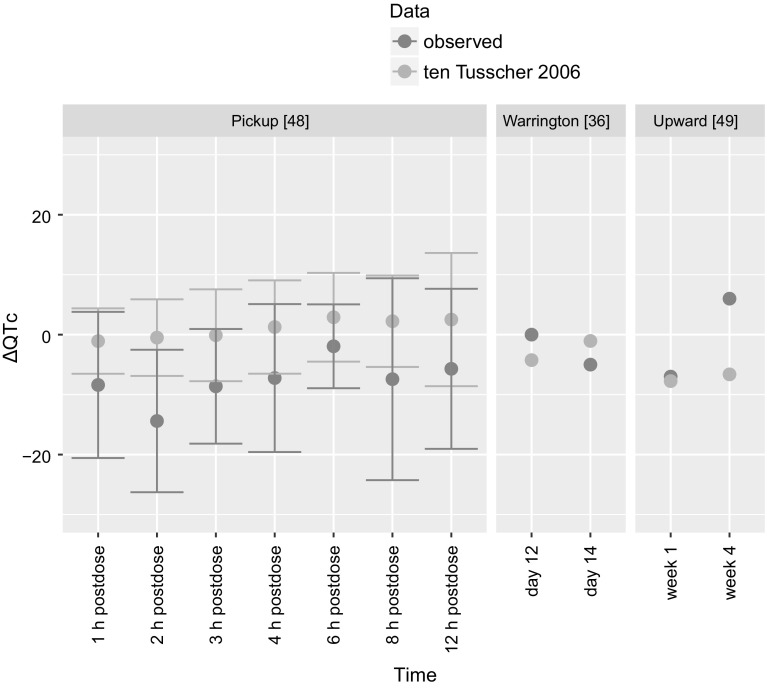
The results of PBPK-QSTS modeling in CSS in ten Tusscher and Panfilov [26] ventricular cardiomyocyte cell model (in blue) compared to clinically observed values (in red) of three clinical trials [36, 48, 49]. The results are presented as mean with standard deviation of ∆QTc (Color figure online)

The effect was expressed as ∆QTc—the difference between QT interval length after drug administration and baseline QT interval length measured in a situation without a drug. The results of PBPK-QSTS modeling compared to observed values are presented in Fig. 5.

In the study by Pickup et al. [48] the mean simulation results of ∆QTc were from − 1.07 ms (1 h postdose) to 2.90 ms (6 h postdose) versus ∆QTc clinically observed from − 14.4 ms (2 h postdose) to − 1.94 ms (6 h postdose). In the study by Warrington et al. [36] the mean simulation results of ∆QTc were − 4.25 ms (in the 12th day of treatment) and − 1.05 ms (in the 14th day of treatment) versus ∆QTc clinically observed: 0 ms (in the 12th day of treatment) and − 5 ms (in the 14th day of treatment). In the study by Upward et al. [49] the mean simulation results of ∆QTc were − 7.75 ms (in the 1st week of treatment) and − 6.62 ms (in the 4th week of treatment) versus ∆QTc clinically observed: − 7 ms (in the 1st week of treatment) and 6 ms (in the 4th week of treatment).

### Model performance: clinical cases of AT intoxication

The PK in 19 cases of AT intoxication was simulated. The simulations’ results are presented as time-concentration profiles of AT and NT in plasma, and in cardiac tissue in Fig. 2 A–P in Supplementary Material. Because of lack of clinically observed precise data on time-matched concentrations of AT and NT, presentation of other PK metrics on the goodness of model prediction was not possible. The results of the cases are described in the order defined in the Materials and Methods section:

1. The predicted mean QTcB in the study mimicking that described by Zakynthinos et al. [43] was 531 ms (520 ms observed in the clinic). In one out of ten simulated patients, arrhythmia was observed. The predicted mean QTcB in the study mimicking that described by Schmidt et al. [35] was 480 ms (517 ms observed in clinic).
2. The predicted QT interval length for AT overdose described by Rudorfer [34] were in the range of 330–373 ms (observed range: 316–438 ms). In eight simulations, 1–2 virtual patients developed arrhythmia (in clinics the arrhythmia on admission was reported in 6 cases).
3. The predicted mean QT in the study mimicking that described by Erdem et al. [50] was 355 ms (400 ms observed). The predicted mean QTcB in the study mimicking that described by Kiyan et al. [51] was 505 ms (521 ms observed in the clinic). The predicted mean QTcB in the study mimicking that described by Paksu et al. [12] was 512 ms (488 ms observed in the clinic). In case of a virtual 25-year-old female with TdP described by Abeyaratne et al. [52], the arrhythmia was simulated in two out of ten virtual patients.

**Fig. 6:**
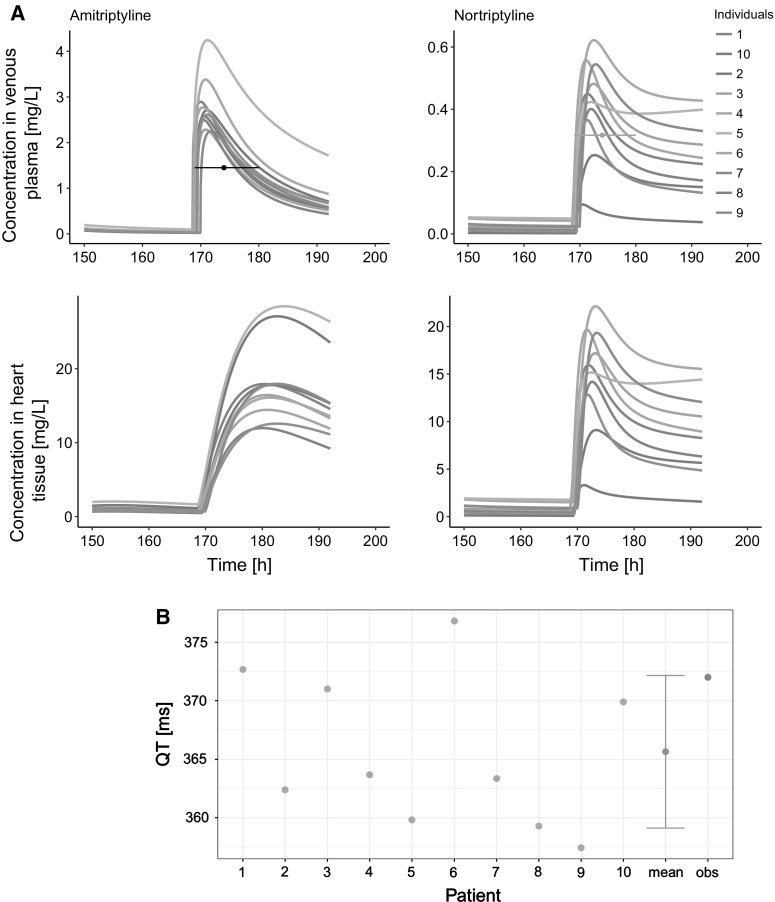
The exemplary individual results of PBPK and PBPK-QSTS modeling in case of 67-year old female intoxicated with 2500 mg of AT [34]. **a** The 1st column contains profiles of AT concentrations, the 2nd has NT concentrations. The 1st row has drug concentrations in venous plasma, the 2nd has drug concentrations in the heart tissue. The black and orange horizontal lines depict the measured concentration of AT, and NT, respectively, and the time interval in which the measurement was conducted. **b** The QT interval length simulated in CSS in ten Tusscher and Panfilov [26] ventricular cardiomyocyte cell model (results of single simulations in green, mean of simulation results in blue) compared to clinically observed value (in orange) (Color figure online)

**Fig. 7:**
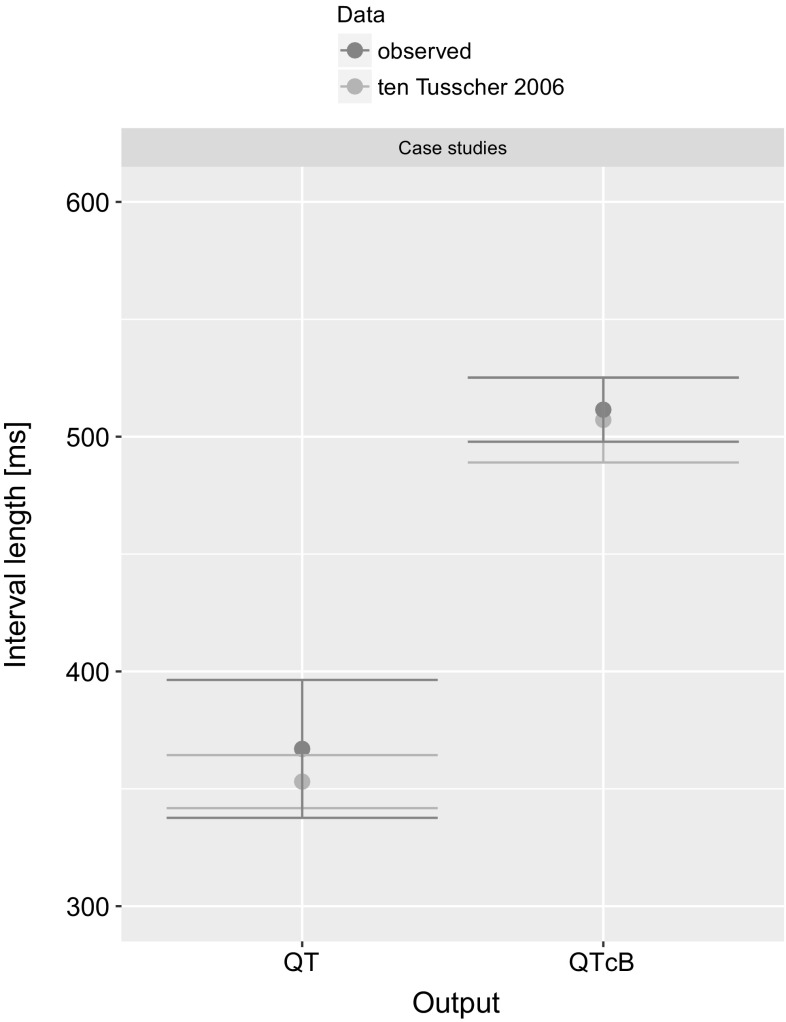
The results of PBPK-QSTS modeling in CSS in ten Tusscher and Panfilov [26] ventricular cardiomyocyte cell model (in blue) compared to clinically observed values (in red). The results are presented as mean with standard deviation of QT or QTcB (Color figure online)

The simulated QT or QTc for each of the clinical cases along with clinical observations are shown in Fig. 3 A–R in Supplementary Material. An exemplary simulation results of individual time-concentration profiles and QT of a 67-year old female intoxicated with 2500 mg of AT are presented in Fig. 6. The mean predicted QT or QTc values from all clinical cases are shown in Fig. 7.

## Discussion

The literature-derived data suggest that there is substantial variability in AT and its metabolite PK observed in the population. It is also the case for the electrophysiological effect of AT, especially in the situation of drug poisoning. We aimed to predict the variability in PK/PD of AT with the use of modeling and simulation paradigm in terms of both, population analysis, and individual cases.

### Modeling the absorption of AT

The basis of the developed system consisted of the recently developed PBPK model for AT and NT established for AT administered intravenously [14]. To keep the model as mechanistic as possible, any of the already established parameters was changed, and the model was extrapolated to the oral route of drug administration. Intestinal absorption is a complex process affected by many drug- and physiology-related factors [70, 71]. The systemically available fraction of an administered dose is the result of the processes occurring in the guts (e.g., metabolism, influx, efflux) and the fraction escaping hepatic first-pass extraction [72]. Mechanistic modeling of gastrointestinal absorption and bioavailability usually requires further segmentation of gastrointestinal track compartment along with its rich parametrization [70, 71, 73, 74]. Our model was developed based on the heterogeneous data derived from various sources, lacking precise information on AT formulation. AT absorption was characterized empirically, assuming first order absorption process. The average values and their distributions of (f_a_ × F_G_) and F for AT were based on observations in humans [15, 16]. (*f*_*a*_×* F*_*g*_*)* was drawn from log-normal distribution of mean 0.832 and coefficient of variation 0.131 [16] without truncation, so the randomly assigned values can reach values larger than 1. It is justified by the enterohepatic circulation that AT is said to undergo [75, 76]. The estimated k_a_ equal 0.24 [h^−1^] and mean *t*_*lag*_ equal 1.33 [h] (assuming CV = 30%) suggest the AT absorption process to be rather slow and delayed in relation to the time of drug ingestion. Although this empirical approach does not give an insight into the physiological aspects of AT absorption, it reflects the net underlying contribution of solubility and dissolution processes, that drug molecules undergo, time of gastric emptying and intestinal transit [72, 77]. Since the parameters were fitted to three trials characterized by different AT doses and different populations, it seemed justified to extrapolate the estimates in further simulations, for the remaining studies.

### Variability in PK of AT in different populations

The PBPK model was capable of providing estimates of the average concentration, the AUC, and the C_max_ within two-fold in most of the simulated trials in terms of both, AT, and its metabolite—NT. The visual inspection (Fig. 1 Supplementary Material) and the dose metrics (Figs. 3, 4) reveal that the model underpredicts the metabolite, i.e., NT concentration. This limitation may be partly explained by the use of simple, minimum-PBPK model to capture PK of the metabolite and not taking into account the NT that is formed during the intestinal metabolic transformation of AT. It is worth noting that none of the model parameters of the previously established model was changed. Because of the scarce data on NT concentration after AT infusion, the model had been fitted to the data derived from only one individual [57]. The model extrapolation to the oral administration of AT in the same individual and another one described in the same source publication was satisfying as the ratio of dose metric met the criterion of two-fold error; it equaled 0.75, and 0.94, respectively (Fig. 3). Probably the estimate of Kpre_NT_ (the tissue to plasma partition coefficient for NT for remaining tissues in the body that were lumped into the ‘rest’ compartment in the minimal PBPK model), although suiting those two cases, does not reflect the average partition coefficient for the population. It can be understandable since the Kps values of NT for different tissues derived from human postmortem data have very wide ranges, for example for the brain the Kp is observed to be in between 5.0 [78] and 37.0 [79], or for the liver from 5.2 [79] to 160.0 [80].

The simulated clinical studies comprised the AT doses in the range from 10 to 100 mg. Besides the margin doses, the PK profiles after other doses were simulated under the scenarios of different populations. The experimentally measured concentrations show a substantial variability in PK after the same doses. For example C_max_ observed after 50 mg of AT given orally varied from 16.7 ng/mL [63] to 50.7 ng/mL [61], which is a three-fold difference. The study by Fagiolino et al. [81] was excluded from the analysis. Although it met the criteria set out in Materials and methods section, it provides the data one order of magnitude higher than in other studies (C_max_ at 606 ng/mL) and a systematic error may be suspected. We were not able to mimic in our model the PK at the lower extreme of observed dose metric: the AUC, C_max_, and the average concentration was overpredicted more than three times in case of the Warrington study [63]. At the upper extreme of observed dose metric there was a study by Jang et al. [61] which was most probably conducted in a Korean population, assuming that the authors affiliation reflects the origin of the subjects taking part in the study. Although our model was parameterized in such a way as to reflect the variability in Caucasian population, its extrapolation to the Asian population appeared to be good enough to capture the AT PKs profiles within two-fold of their respective metrics [53, 56, 61]. Another source of variability in PK comes from polymorphism in the enzymes engaged in the drug metabolism [82, 83]. In most of the mimicked studies the participants did not have the CYPs phenotype assessed with exception of two trials [20, 64]. In Venkatakrishnan’s [20] study only one subject was determined as CYP2D6 poor metabolizer. The others were CYP2D6 and CYP2C19 extensive metabolizers. In the trial carried out by Balant-Gorgia et al. [64] the ratio of number of poor hydroxylators to extensive hydroxylators was 3: 4. Although our PBPK model does not allow for CYPs phenotype determination, the prediction of AT PK profile met the criteria of being within two-fold error ranges. However, the NT PK profile was underpredicted over 2.5 times. Being aware of the model’s limitations it can be concluded that its predictions are good enough to make use of the model’s capability of predicting free drug cardiac concentrations which is suggested to trigger cardiac effect [84]. The PBPK model performance verification was done for single as well as multiple therapeutic doses of AT in different populations.

### PBPK: QSTS at the population level

AT has been on the market for more than 50 years and is still frequently prescribed [85]. However, there is QT interval prolongation listed among its side effects and according to CredibleMeds [86] it is classified as “drug with conditional TdP risk”. The conditions that predispose subject to drug-related TdP are as follows: bradycardia, low serum potassium or magnesium level, excessive dose, impaired drug elimination, and drug PD interaction. The main AT metabolite—NT—has been assigned to category of “drugs with possible TdP risk”. According to the ICH E14 guidance [87] on drug cardiac safety, the evaluated endpoint that the regulators are concerned about is QT/QTc prolongation exceeding 5 ms, as judged by whether the upper bound of the 95% confidence interval around the mean effect on QTc exceeds 10 ms. Under the normal conditions and in therapeutic doses, AT should not put one at risk of TdP arrhythmia and the clinical trials on AT confirm the AT cardiac safety. We have confirmed that observations in numerical experiment with the in silico realised PBPK-QSTS model. The use of the predicted free cardiac AT and NT concentrations as input in ten Tusscher [26] model implemented in CSS allowed for confirmation of no effect of AT used in therapeutic doses on QT/QTc in either healthy individuals or at least with no physical illness. The model turned out to be capable of predicting AT cardiac safety under different scenarios (single/multiple dose) and in different time scales: hourly -, daily-, and weekly time scale. The simulated mean ∆QTc did not exceed 5 ms in neither of the assessed time points, likewise in the clinical trials. There was only one exceptional observation, i.e., the difference between mean QTc measured in the 4th week of AT treatment and the mean QTc at baseline equaled 6 ms in the study by Upward et al. [49]. However, since that trial was conducted in the 1980’s, it was not designed according to the current ICH E14 guidelines and the results allowed the authors for the conclusion that “the QTc was not significantly altered”. When discussing the results the authors pointed out the AT-related increase in heart rate, which was not taken into account in case of therapeutic concentrations, which may cause the difference between the simulation and the observation in that time point for this particular study. When following the study by Pickup et al. [48], in which the information on precise time of the day of ECG assessment was provided, in CSS simulation we considered not only inter-individual- but also intra-individual variability which results from circadian rhythms in heart rate and ion concentrations [88, 89]. Thus, the trends in ∆QTc values observed by Pickup et al. were confirmed: the higher ∆QTc occurred in the afternoon with the highest value observed (and simulated as well) 6 h postdose. The only difference was for the trend of the ∆QTc values in 1 and 2 h postdose.

### PBPK: QSTS model applied to individuals

We went a step further from population PBPK-QSTS analysis towards so called ‘personalized medicine’ and simulation of drug adverse reactions in ‘virtual twins’ [8] based on the established PBPK model structure [14] and ventricular cardiomyocyte cell model [26], both accounting for inter-individual variability. Although personalized therapy was not the aim of the study, the developed PBPK model has a capability of being used for this purpose. Modeling the individual cases of AT intoxication proving the model to be functional, should be viewed as the first step in personalizing the treatment via modeling and simulation approach, Since very detailed data on patient’s characteristic were lacking, we stuck to the patient’s age, sex, previous AT treatment, heart rate, potassium, sodium, and calcium concentrations, if available. Due to the specific character of simulated cases, i.e., AT intoxications, the ingested dose and time of AT ingestion were estimated approximately by the clinicians, and the measurements of drugs concentrations were provided only in one time point. Therefore, the assessment of goodness of model prediction other than visual inspection was not possible. The extrapolation of our PBPK model to toxic doses was preceded by the model verification in case of therapeutic ranges of AT levels. Only those studies in which most probably no other drugs besides AT were taken by the patients were chosen. The only one exception was the case described by Schmidt et al. [35], where along with AT, tilidine, lorazepam, and ethanol were known to be taken. However neither of these substances is on the CredibleMeds list [86], so no PD interaction was assumed, and measured instead of the simulated AT and NT concentrations were used directly to calculate free cardiac concentration (1st group of cases described in the Materials and Methods section).

Regarding the 2nd group of cases described in the Materials and Methods section, the PBPK model predictions matched or were very close to the measured concentrations (Supplementary Material Fig. 2) despite the inaccurate clinical data, which confirmed PBPK the model’s feasibility. Free cardiac concentrations used as the driving force for the simulation of the pseudo-ECG traces gave good results, as judged by comparison of means of predicted and observed QT or QTcB length (Fig. 5). In most cases, the overdosed patients suffered from tachycardia, so Bazett correction of QT interval length for heart rate was unjustified [90]. It was used only if such an ECG parameter was provided in the source paper.

It seems that AT in supratherapeutic doses more frequently poses an effect on the heart rate rather than on QT interval length [28, 34]. That observation led to the E_max_ model development which binds the RR interval length with AT plasma concentration. The E_max_ model was used when mimicking the case study reported by Paksu et al. [12]—case no. 26 in the source publication, for which the total estimated dose (750 mg) was provided. The observed QTc length (488 ms) were in between the minimal (481.6 ms) and maximal (533.7 ms) value of QTc simulated for that case (Supplementary Material, Fig. 3O). It showed that E_max_ model worked in practice. It is worth adding that in some cases [12, 34] the arrhythmia was reported. In our simulations often one to two out of ten virtual patients poisoned with AT developed arrhythmia. Since all of ten simulations, for each of the mimicked cases, were run with the same settings regarding AT and NT concentrations and heart rate, there were other patient specific parameters that mattered and predisposed the virtual individuals to electrophysiology disruptions. Indeed, the severity of clinical findings of AT intoxication is weakly correlated with AT serum levels [12]. Abeyaratne et al. [52] described the case of 25-year old female intoxicated with ca. 500 mg of AT who developed TdP arrhythmia 2 h after poisoning. 500 mg is much smaller dose than in other reports, for example 3000 mg [34] or almost 4000 mg [50] which were not associated in those specific cases with arrhythmia occurrence. In Abeyaratne’s study simulation the TdP in 2 out of 10 virtual individuals was repeated. The feature that distinguished that case from the others is the fastest heart rhythm (165 bpm). The ion concentrations of the patient were unknown and assumed to be normal. Detailed knowledge of patient’s biochemical parameters should improve the PD predictivity.

There are other cases of AT-related TdP described in the literature which were not simulated because of either lacking information on AT dose or concentration [91], pediatric case [92] or co-medication (fluconazole [93], loperamide [94]) that may pose an effect on cardiac electrophysiology. Because those cases are the examples of arrhythmia not only related to TCA overdose, they support the thesis that the drug triggered cardiotoxicity is a complex process, and many internal, as well as external factors, should be taken into account in model-based drug safety assessment. It seems justified to state that verified PBPK-QSTS models can be of help for the population and individuals safety assessment.

## Conclusions

The herein described PBPK model allows for AT and NT free cardiac concentration predictions. The model was verified in terms of PK and the usefulness of predicted cardiac concentrations for AT-related electrophysiology effect modeling as well. Detailed mechanistic models which have the ability to predict between-subject variability have the potential of PK or PK/PD assessment in population, as well as in certain individuals providing that patient-specific information is available. The results of our study support the validity and feasibility of the PBPK-QSTS modeling development for personalized medicine.

## Electronic supplementary material

Below is the link to the electronic supplementary material.

Supplementary material 1 (PDF 6007 kb)
