# Supplementary material for: Physiologically based pharmacokinetic-quantitative systems toxicology and safety (PBPK-QSTS) modeling approach applied to predict the variability of amitriptyline pharmacokinetics and cardiac safety in populations and in individuals
Source: J Pharmacokinet Pharmacodyn. 2018 Jun 25;45(5):663–77. doi: 10.1007/s10928-018-9597-6 (PMC6182726; doi:10.1007/s10928-018-9597-6)

Physiologically Based Pharmacokinetic – Quantitative Systems Toxicology and Safety (PBPK-QSTS) Modeling Approach Applied to Predict the Variability of Amitriptyline Pharmacokinetics and Cardiac Safety in Populations and in Individuals.

Zofia Tylutki*[1], Aleksander Mendyk[2], and Sebastian Polak[1,3]

1. Unit of Pharmacoepidemiology and Pharmacoeconomics, Department of Social Pharmacy, Faculty of Pharmacy, Jagiellonian University Medical College, Medyczna 9 Str., 30-688 Krakow, Poland

2. Department of Pharmaceutical Technology and Biopharmaceutics, Jagiellonian University Medical College, Medyczna 9 St., 30-688 Krakow, Poland

3. Certara-Simcyp, Level 2-Acero, 1 Concourse Way, Sheffield, S1 2BJ, United Kingdom

*Corresponding author: Zofia Tylutki, zofia.tylutki@doctoral.uj.edu.pl

		PBPK model parameters						PD model parameters										
								In vitro ion channel inhibition				Ionic concentration						
Study	Purpose	Daily dose (AT chl) [mg]	Dosing schedule	Number of males	Nubmer of females	Age	seed	IC50 IKr (AT)	IC50 INa (AT)	IC50 ICa (AT)	IC50 IKr (NT)	[K+] [mM]	[Na+] [mM]	[Ca2+] [mM]	Number of studies/ Individuals per study	RR [ms]	seed	PD output
Curry 1985 [1]	Model optimization	25	single	10	0	21 – 40	1111											
Venkatakrishnan 2001 [2]		50	single	7	1	24 – 45												
Gupta 1999 [3]		75	single	15	0	21 -37												
Nam 2015 [4]	PBPK model performance verification	10	single	12	0	19 - 50												
		25	single	12	0	19 - 50												
Dorian 1983 [5]		25	single	2	3	23 – 29												
Liedholm 1998 [6]		25	single	0	9	25 – 54												
Ogura 1983 [7]		25	single	7	0	21 – 25												
		25	single	7	0	65 – 74												
Bhatt 2010 [8]		50	single	24	0	18 – 60												
Burch 1981 [9]		50	single	4	2	18 – 40												
Ghahramani 1996 [10]		50	single	2	0	18 – 40												
Jang 1990 [11]		50	single	6	0	25 – 29												
Mellstorm 1983 [12]		50	single	5	4	25 – 49												
Warrington 1984 [13]		50	single	2	4	19 – 22												
Balant-Gorgia 1982 [14]		75	single	6	1	22 – 29												

Burch 1981 [9]		100	single	4	2	18 – 40												
Baumann 1986 [15]		75, 150	multiple	9	21	20 – 60												
Gupta 1998 [16]		75	multiple	16	8	19 - 49												
Gupta 1999 [3]		25	multiple	15	0	21 – 37												
Miljković 1996 [17]		150	multiple	3	3	31 - 59												
		75	multiple	3	6	39 – 56												
Schulz 1983 [18]		80	single	1	0	21												
		40	single	1	0	70												
Vandel 1993 [19]		125	multiple	7	10	24 – 69												
Pickup 1982 [20]	PBPK-QSTS	75	single	8	0	20 -28		1.801 [21]	3.5 [22]	21.9 [22]	2.27 [23]	CSS default	CSS default	CSS default	10 / 8	default	1 / 2*	QTcBi
Upward 1988 [24]		150, 200	multiple	5	7	29 – 61									10 / 12	default	1	QTcB
Warrington 1989 [25]		75	multiple	12	0	20 -30									10 / 12	default		ΔQTI
Zakynthinos 2000 [26]	PD model performance verification			0	1	44									10 / 1	480		QTcB
Schmidt 2015 [27]				0	1	54										638		
Rudorfer 1982 [28]	PBPK-QSTS	7500	multiple	0	1	29	1111									462		QT
		3600	single	1	0	25										429		
		750	multiple	0	1	29										429		
		2500	multiple	0	1	67										517		
		350	single	0	1	19										500		

		1500	single	1	0	22										484		
		375	single	0	1	23										429		
		3000	single	0	1	39										400		
		1750	single	0	1	32										500		
		2000	multiple	1	0	25										556		
		1500	multiple	1	0	17										480		
		3000	single	0	1	19										476		
Erdem 2010 [29]		3750	multiple	1	0	24						2.9	136			429		QT
Kiyan 2006 [30]		300	multiple	0	1	33						CSS default	CSS default			500		QTcB
Paksu 2014 [31]		750	multiple	1	0	34							137			500**		QTcB
Abeyaratne 2016 [32]		500	single	0	1	25							CSS default			364		TdP occurence

*40 individuals were simulated with the seed 1, and 40 with the seed 2; the file had to be divided because of technical issues, **There was no data on RR interval length in the publication. The individual RR were predicted in the E_{max} model. The mean of model-predicted RR was used as input value in CSS.

Table 1: The details of simulation scenarios and settings.

References to the table:

1. Curry SH, DeVane CL, Wolfe MM (1985) Cimetidine Interaction with Amitriptyline. Eur J Clin Pharmacol 29:429–433

2. Venkatakrishnan K, Schmider J, Harmatz J, Ehrenberg B, von Moltke L, Graf J, Mertzanis P, Corbett K, Rodriguez M, Shader R, Greenblatt D (2001) Relative Contribution of CYP3A to Amitriptyline Clearance in Humans : In Vitro and In Vivo Studies. J Clin Pharmacol 41:1043–1054

3. Gupta SK, Shah JC, Hwang SS (1999) Pharmacokinetic and pharmacodynamic characterization of OROS and immediate-release amitriptyline. J Clin Pharmacol 48:71–78

4. Nam Y, Lim CH, Lee HS, Chung SJ, Chung YH, Shin YK, Kim MG, Sohn UD, Kim HC, Jeong JH (2015) Single-dose, Randomized, Open-label, 2-way Crossover Study of the Pharmacokinetics of Amitriptyline Hydrochloride 10- and 25-mg Tablet in Healthy Male Korean Volunteers. Clin Ther 37:302–310 . doi: 10.1016/j.clinthera.2014.09.010

5. Dorian P, Sellers EM, Reed KL, Warsh JJ, Hamilton C, Kaplan HL, Fan T (1983) Amitriptyline and Ethanol: Pharmacokinetic and Pharmacodynamic Interaction. Eur J Clin Pharmacol 25:325–331 . doi: 10.1007/BF01037943

6. Liedholm H, Lidén A (1998) Food intake and the presystemic metabolism of single doses of amitriptyline and nortriptyline. Fundam Clin Pharmacol 12:636–642 . doi: 10.1111/j.1472-8206.1998.tb00998.x

7. Ogura C, Kishimoto A, Mizukawa R, Hazama H, Honma H, Kawahara K (1983) Age Differences in Effects on Blood Pressure, Flicker Fusion Frequency, Salivation and Pharmacokinetics of Single Oral Doses of Dothiepin and Amitriptyline. Eur J Clin Pharmacol 25:811–814 . doi: 10.1007/BF00542525

8. Bhatt M, Shah S, Shivprakash (2010) Development and validation of amitriptyline and its metabolite in human plasma by ultra performance liquid chromatography-tandem mass spectrometry and its application to a bioequivalence study. Biomed Chromatogr 24:1247–1254 . doi: 10.1002/bmc.1435

9. Burch JE, Hullin RP (1981) Amitriptyline pharmacokinetics. A crossover study with single doses of amitriptyline and nortriptyline. Psychopharmacology (Berl) 74:35–42 . doi: 10.1007/BF00431754

10. Ghahramani P, Lennard MS (1996) Quantitative analysis of amitriptyline and nortriptyline in human plasma and liver microsomal preparations by high-performance liquid chromatography. J Chromatogr B Biomed Appl 685:307–313 . doi: 10.1016/S0378-4347(96)00193-4

11. Jang I-J, Shin J-G, Shin S-G, Park C-W, Kim J-J, Woo J-I, Chao I-J (1990) Pharmacokinetics of Amitriptyline Demethylation ; A Crossover Study with Single Doses of Amitriptyline and Nortriptyline. Seoul J Med 31:11–18

12. Mellstrom B, Bertilsson L, Lou Y-C, Sawe J, Sjoqvist F (1983) Amitriptyline metabolism: Relationship to polymorphic debrisoquine hydroxylation. Clin Pharmacol Ther 34:516–520 . doi: 10.1038/clpt.1983.207

13. Warrington SJ, Ankier SI, Turner P (1984) An evaluation of possible interactions between ethanol and trazodone or amitriptyline. Br J Clin Pharmacol 18:549–557

14. Balant-Gorgia AE, Schulz P, Dayer L, Balant L, Kubli A, Gertsch C, Garrone G (1982) Role of oxidation polymorphism on blood and urine concentrations of amitriptyline and its metabolites in man. Arch Psychiatry Neurol Sci 232:215–222 . doi: 10.1007/BF02141782

15. Baumann P, Jonzier-Perey M, Koeb L, Le P, Tinguely D, Schopf J (1986) Amitriptyline Pharmacokinetics and Clinical Response: I. Free and Total Plasma Amitriptyline and Nortriptyline. Int Clin Psychopharmacol 1:89–101

16. Gupta SK, Shah J, Guinta D, Hwang S (1998) Multiple-Dose Pharmacokinetics Pharmacodynamics of OROS and Immediate-Release Amitriptyline Hydrochloride Formulations. J Clin Pharmacol 38:60–67

17. Miljkovic B, Pokrajac M, Timotijevic I, Varagic V (1996) Clinical response and plasma concentrations of amitriptyline and its metabolite-nortriptyline in depressive patients. Eur J Drug Metab Pharmacokinet 21:251–255

18. Schulz P, Turner-Tamiyasu K, Smith G, Giacomini KM, Blaschke TF (1983) Amitriptyline disposition in young and elderly normal men. Clin Pharmacol Ther 33:360–366 . doi: 10.1038/clpt.1983.46

19. Vandel S, Bertschy G, Perault MC, Sandoz M, Bouquet S, Chakroun R, Guibert S, Vandel B (1993) Minor and clinically non-significant interaction between toloxatone and amitriptyline. Eur J Clin Pharmacol 44:97–99 . doi: 10.1007/BF00315289

20. Pickup A, Bojanowski L, Dawling S, Dinsdale J, Gosling R (1982) Comparison of the Effect of Amitriptyline in Standard and Sustained-Release Formulations on Cardiac Systolic Time Intervals. J Cardiovasc Pharmacol 4:575–583

21. Guo L, Guthrie H (2005) Automated electrophysiology in the preclinical evaluation of drugs for potential QT prolongation. J Pharmacol Toxicol Methods 52:123–135 . doi: 10.1016/j.vascn.2005.04.002

22. Cao X, Lee YT, Holmqvist M, Lin Y, Ni Y, Mikhailov D, Zhang H, Hogan C, Zhou L, Lu Q, Digan ME, Urban L, Erdemli G (2010) Cardiac Ion Channel Safety Profiling on the IonWorks Quattro Automated Patch Clamp System. Assay Drug Develompment Technol 8:766–780 . doi: 10.1089/adt.2010.0333

23. Jeon S-H, Jaekal J, Lee SH, Choi B-H, Kim K-S, Jeong H-S, Han SY, Kim E-J (2011) Effects of nortriptyline on QT prolongation : A safety pharmacology study. Hum Exp Toxicol 30:1649–1656 . doi: 10.1177/0960327110396528

24. Upward J, Edwards J, Goldie A, Waller D (1988) Comparative effects of fluoxetine and amitriptyline on cardiac function. Br J Clin Pharmacol 26:399–402

25. Warrington SJ, Turner P, Skrumsager BK (1989) Cardiovascular (ECG and systolic time intervals) and anticholinergic effects of repeated doses of femoxetine - a comparison with amitryptyline and placebo in healthy men. Br J Clin Pharmacol 27:343–351

26. Zakynthinos E, Vassilakopoulos T, Roussos C, Zakynthinos S (2000) Abnormal atrial and ventricular repolarisation resembling myocardial injury after tricyclic antidepressant drug intoxication. Heart 83:353–354

27. Schmidt JJ, Bertram A, Kühn-Velten WN, Suhling H, Wiesner O, Schneider A, Kielstein JT (2015) Treatment of amitriptyline intoxications by extended high cut-off dialysis. Clin Kidney J 8:796–799 . doi: 10.1093/ckj/sfv094

28. Rudorfer M V (1982) Cardiovascular Changes and Plasma Drug Levels after Amitriptyline Overdose. J Toxicol Clin Toxicol 19:67–78

29. Erdem D, Akan B, Albayrak MD, Üstünbaş P, Ugiş C, Gögüs N (2010) Plasmapheresis Application in High-Dose Amitriptyline Intoxication. Eur J Surg Sci 1:58–62

30. Kiyan S, Aksay E, Yanturali S, Atilla R, Ersel M (2006) Acute Myocardial Infarction Associated with Amitriptyline Overdose. Pharmacol Toxicol 98:462–466

31. Paksu S, Duran L, Altuntas M, Zengin H, Salis O, Ozsevik SN, Albayrak H, Murat N, Guzel A, Paksu MS (2014) Amitriptyline overdose in emergency department of university hospital: Evaluation of 250 patients. Hum Exp Toxicol 33:980–990 . doi: 10.1177/0960327113520019

32. Abeyaratne DDK, Liyanapathirana C, Gamage A, Karunarathne P (2016) Survival after severe amitriptyline poisoning with prolonged ventricular tachycardia and cardiac arrest. BMC Res Notes 1–5 . doi: 10.1186/s13104-016-1963-0

Figure 1: The simulated PK profiles along with AT and NT concentrations observed in clinic

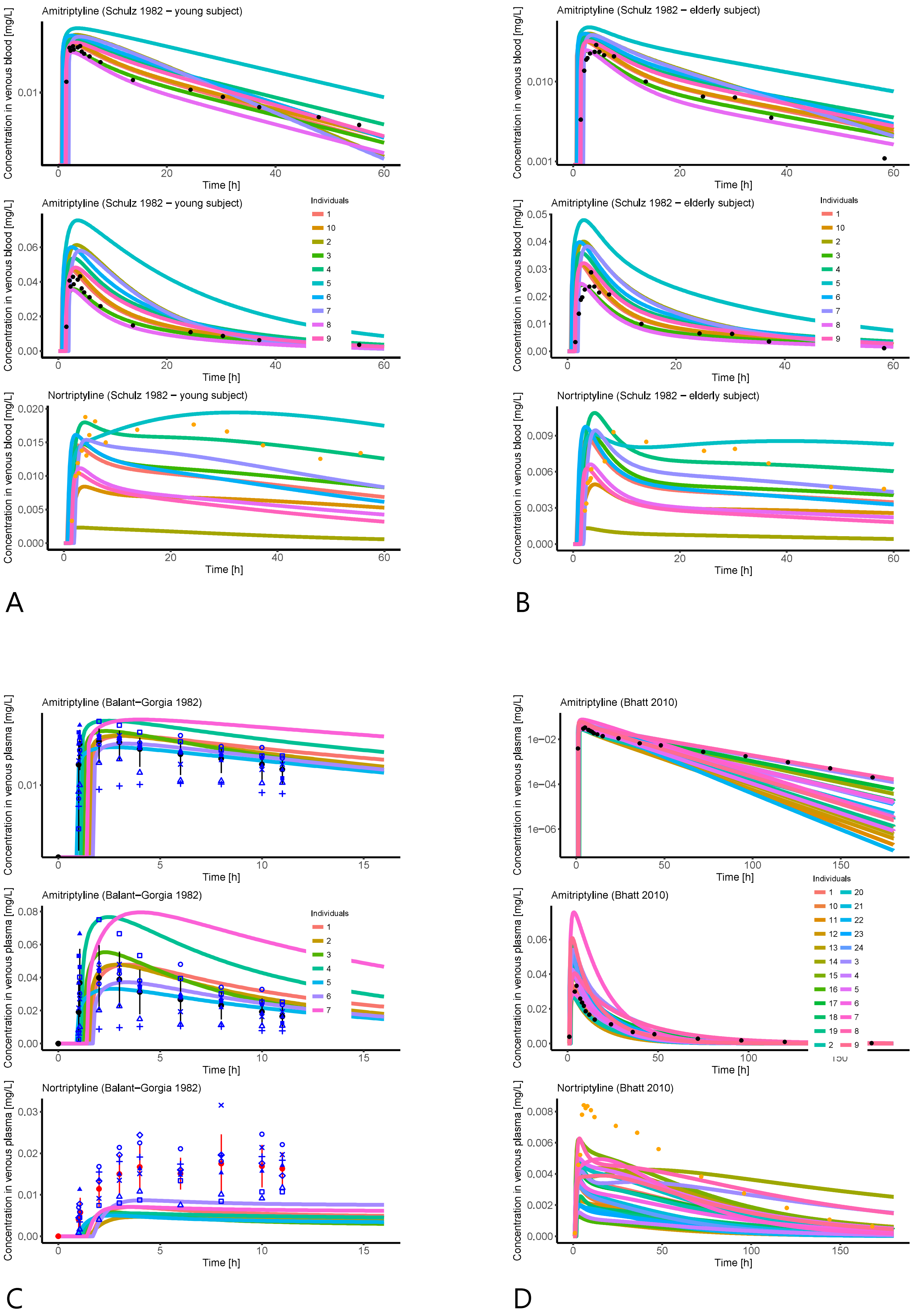

Figure 1 - Continued

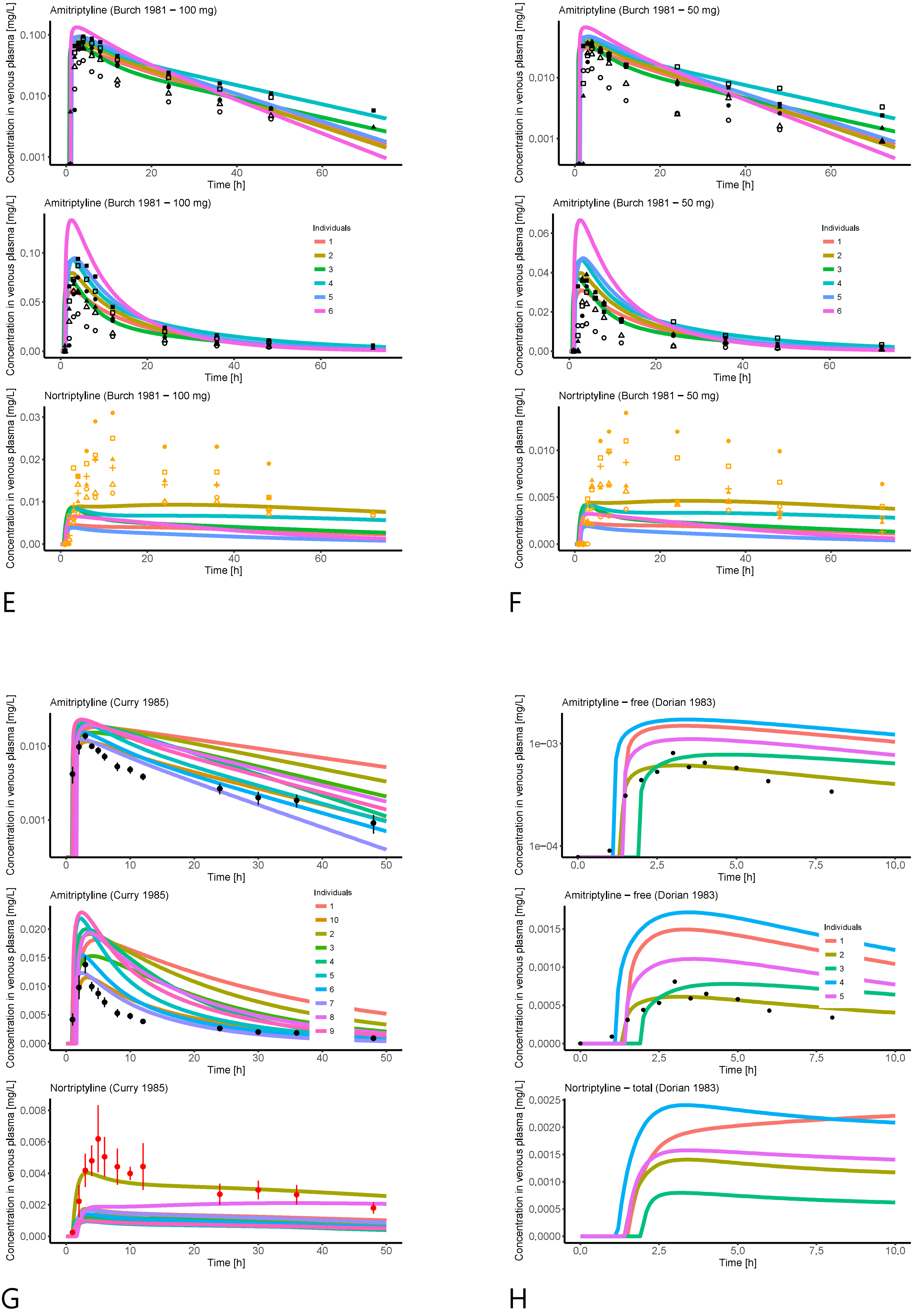

Figure 1 - Continued

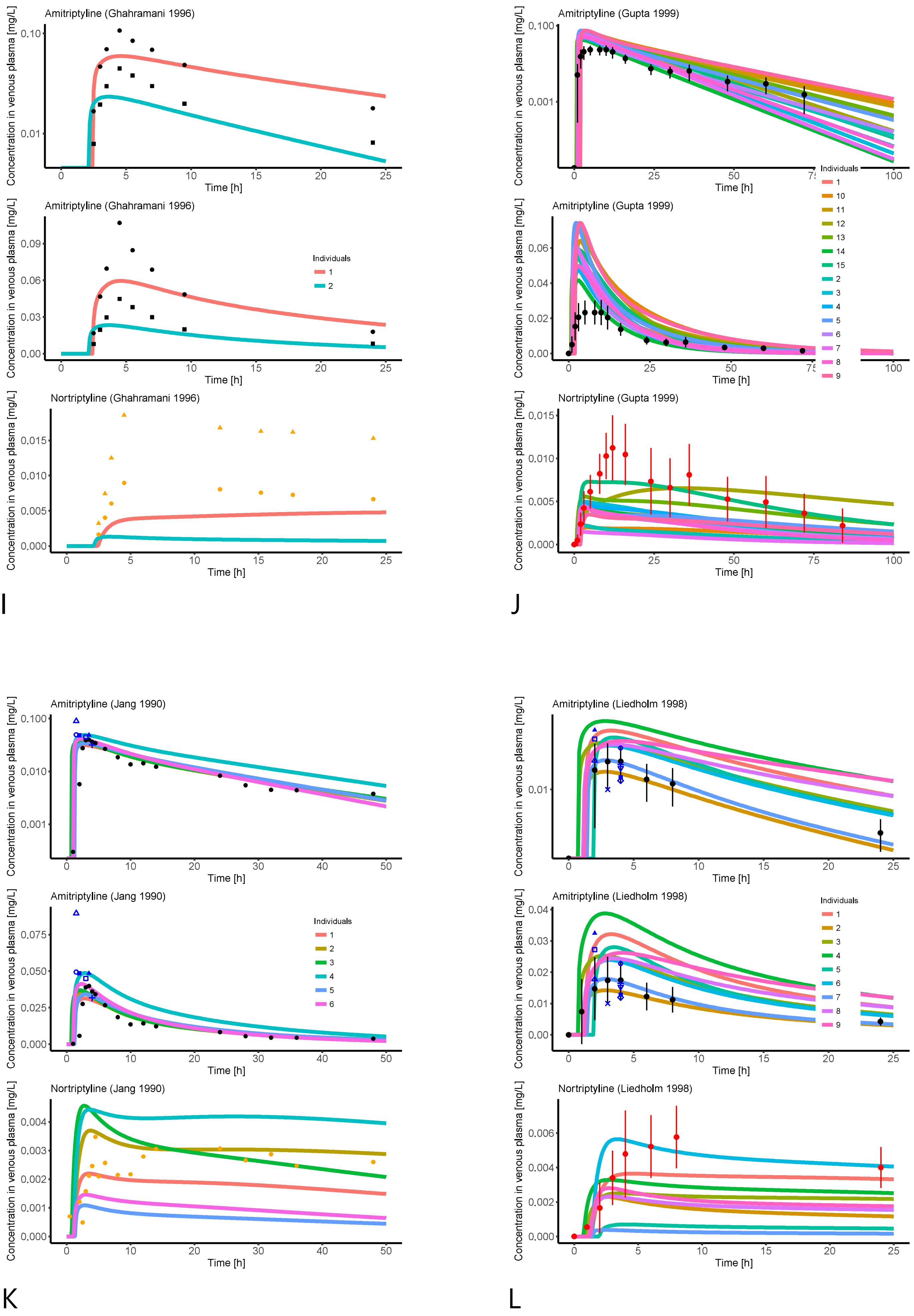

Figure 1 - Continued

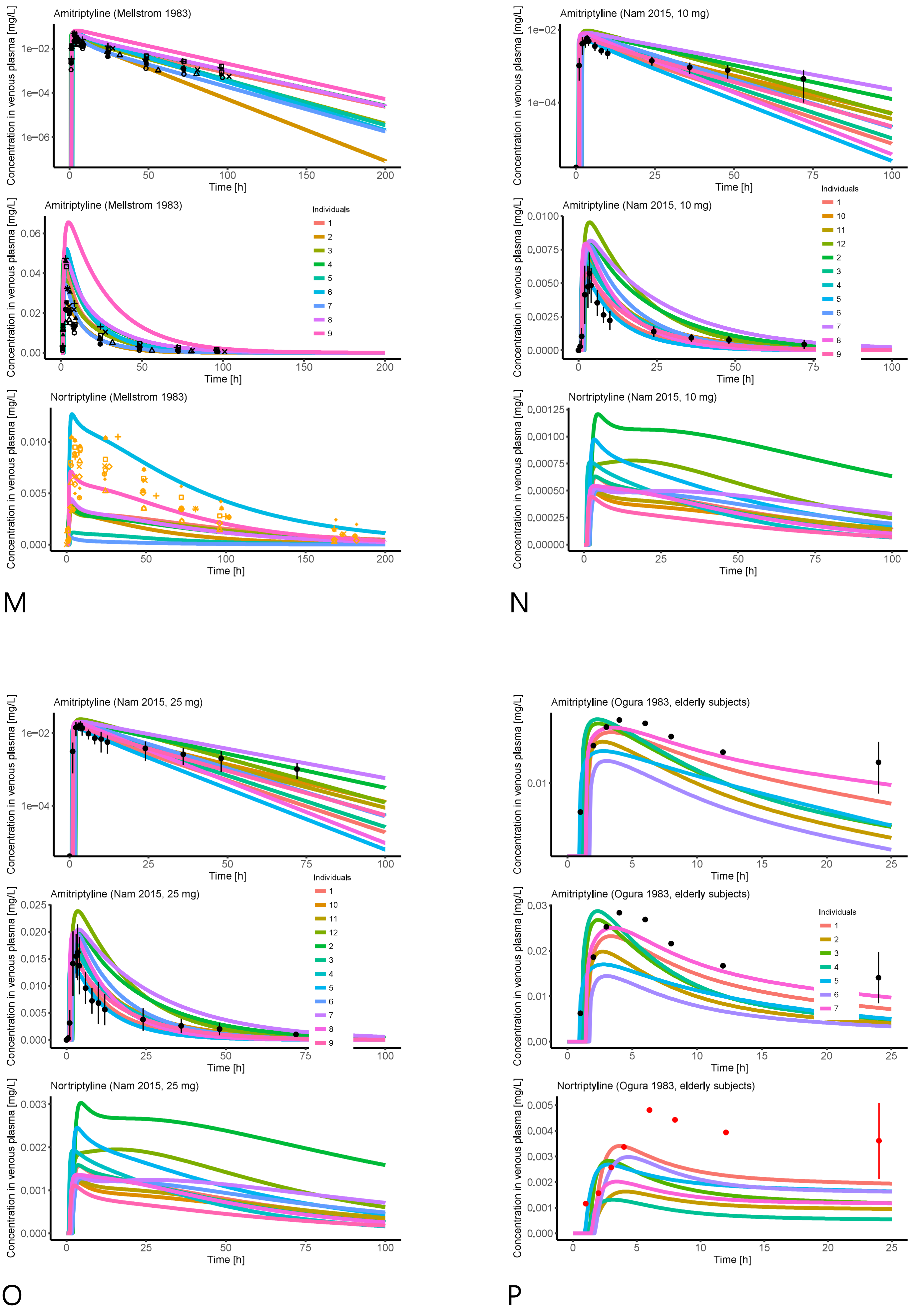

Figure 1 - Continued

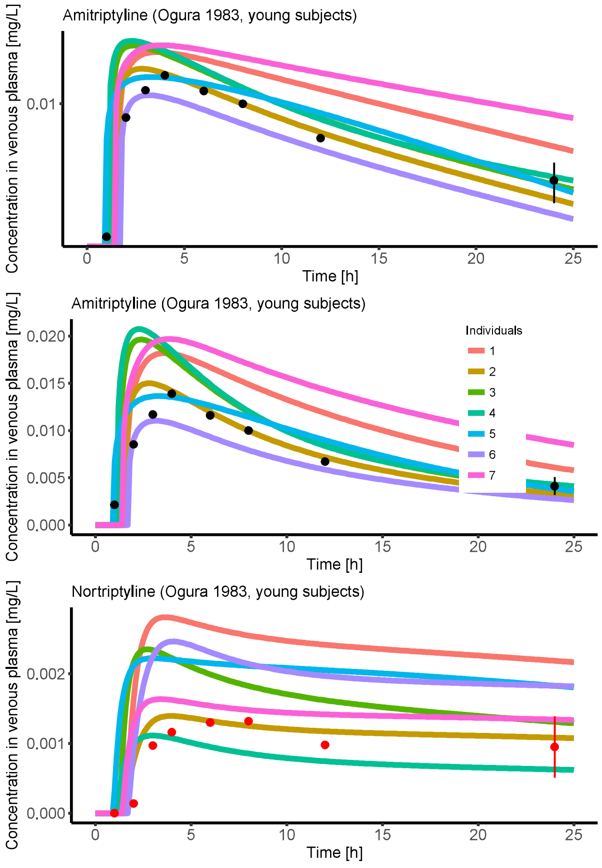

Q

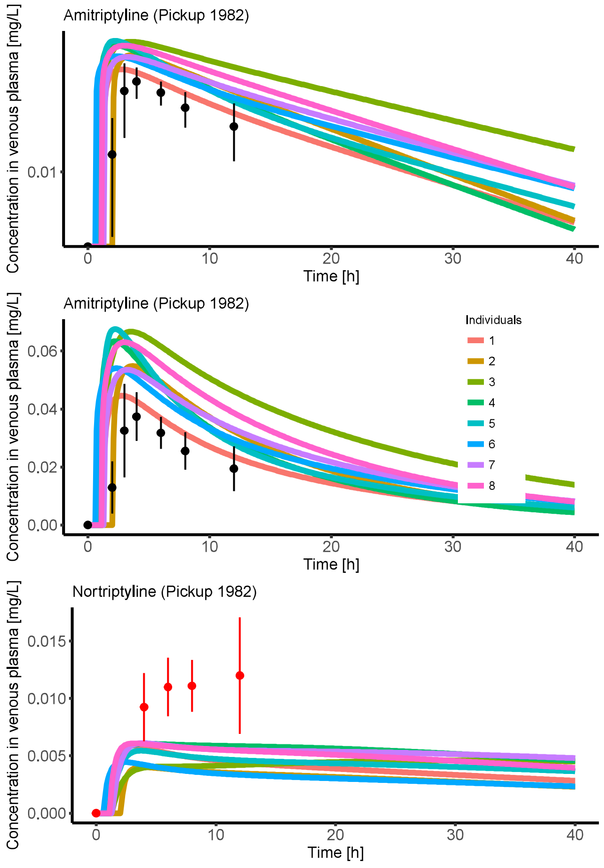

R

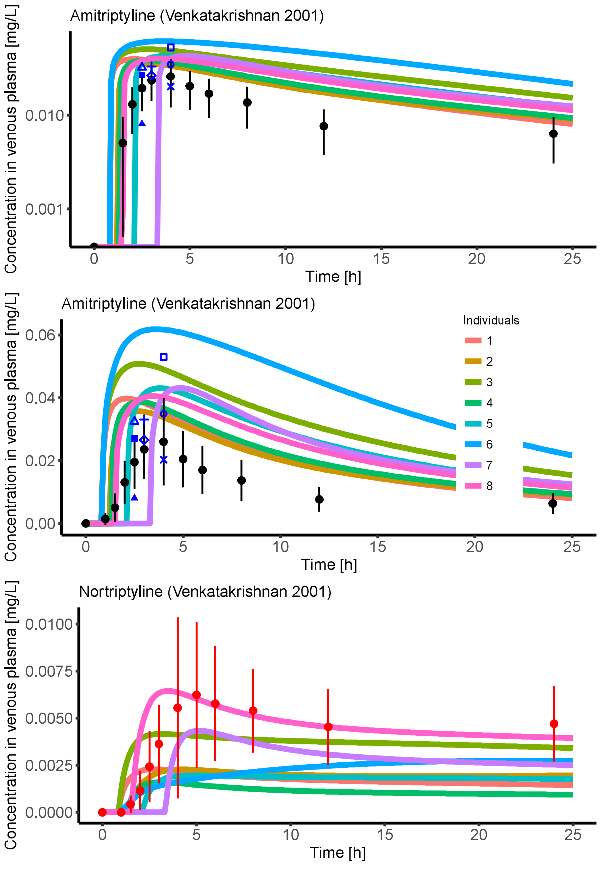

S

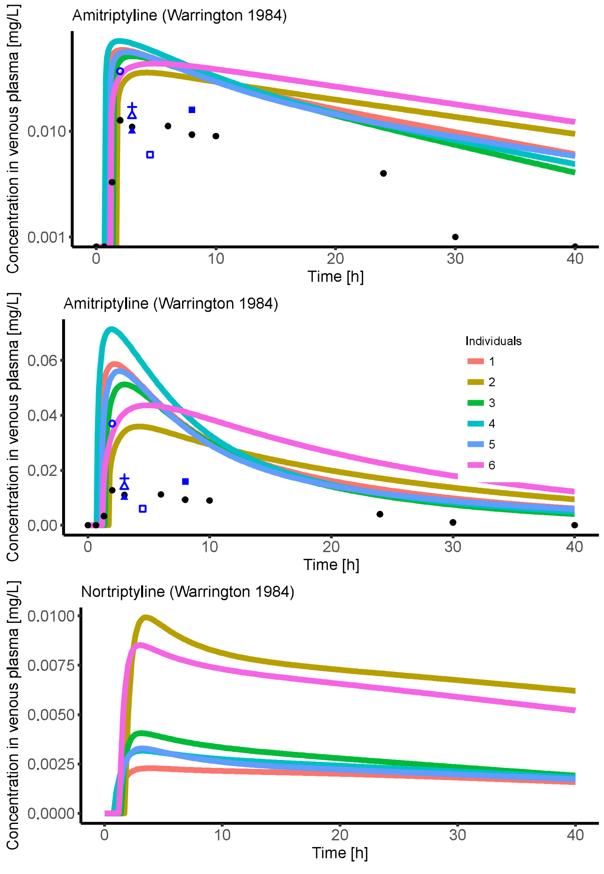

T

Figure 1 - Continued

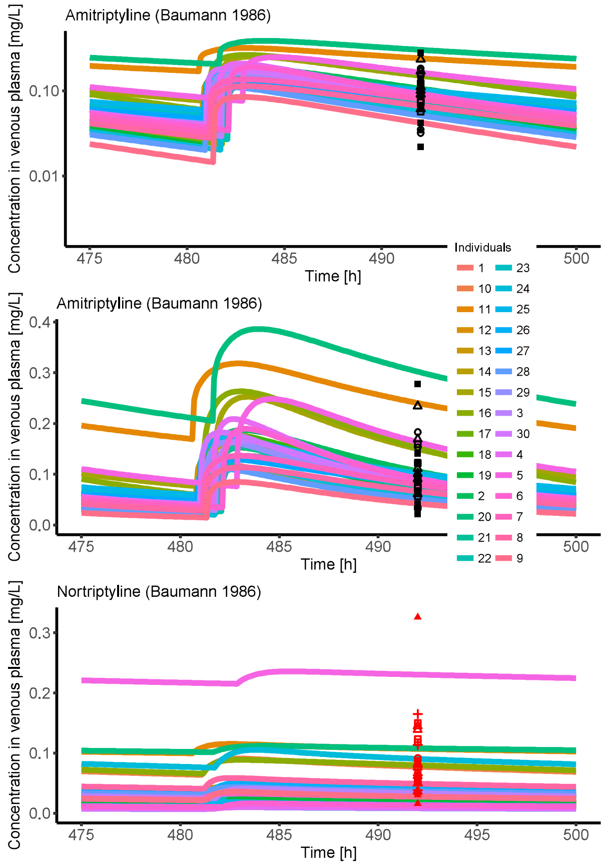

U

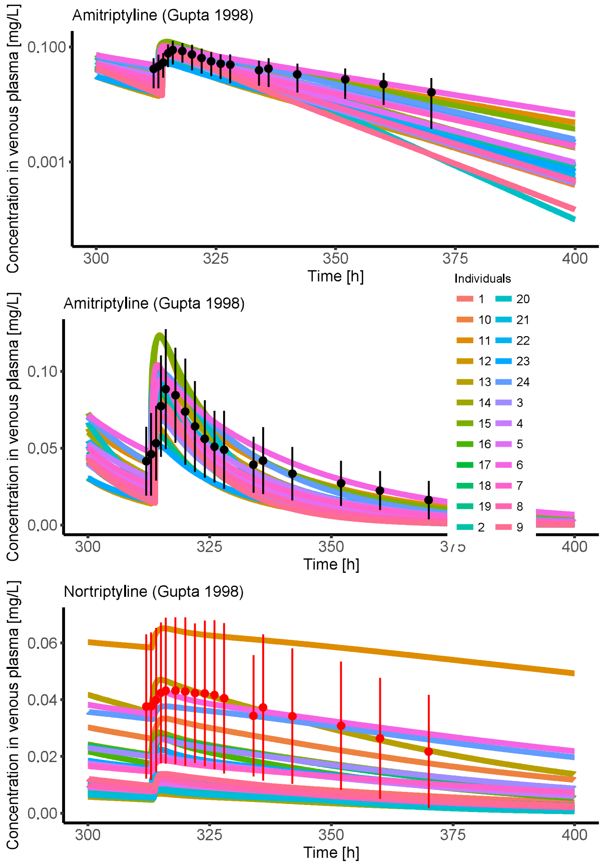

V

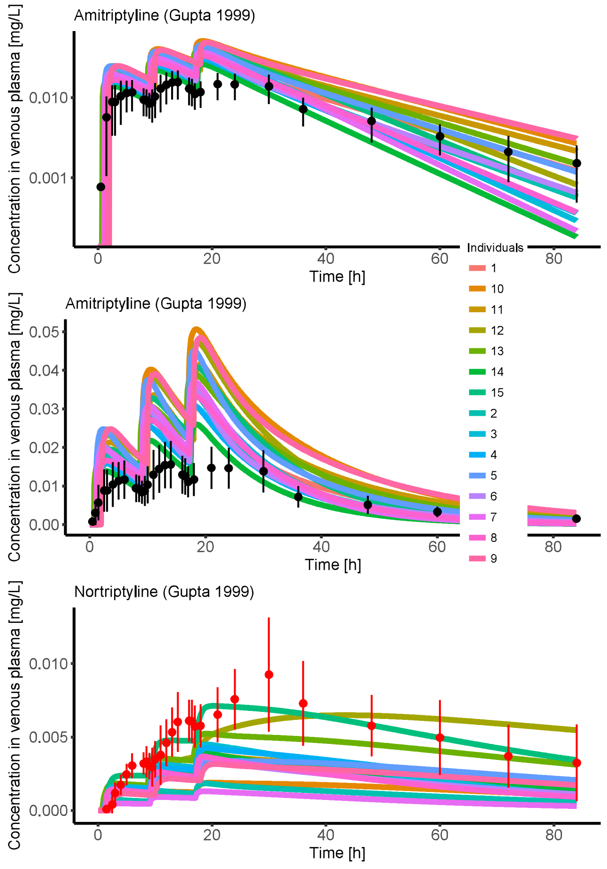

W

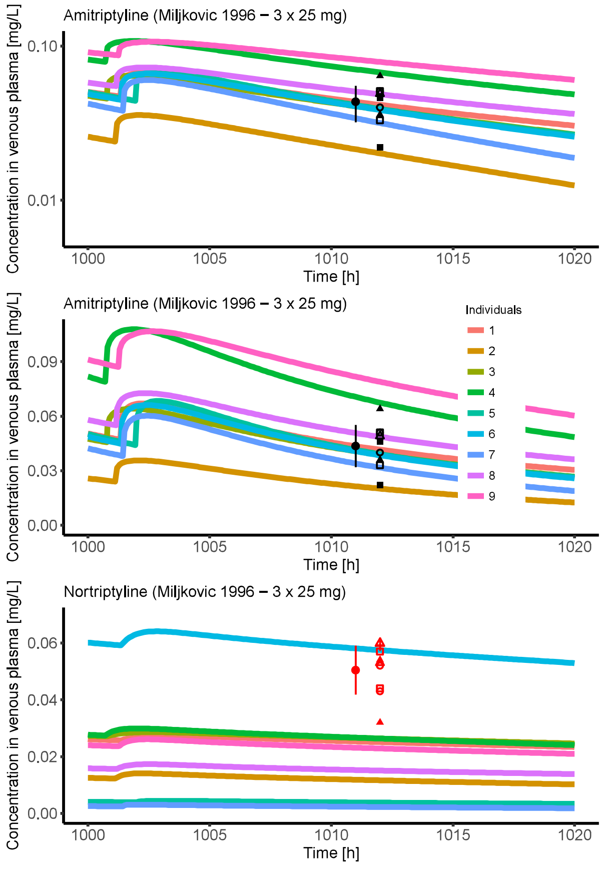

X

Figure 1 - Continued

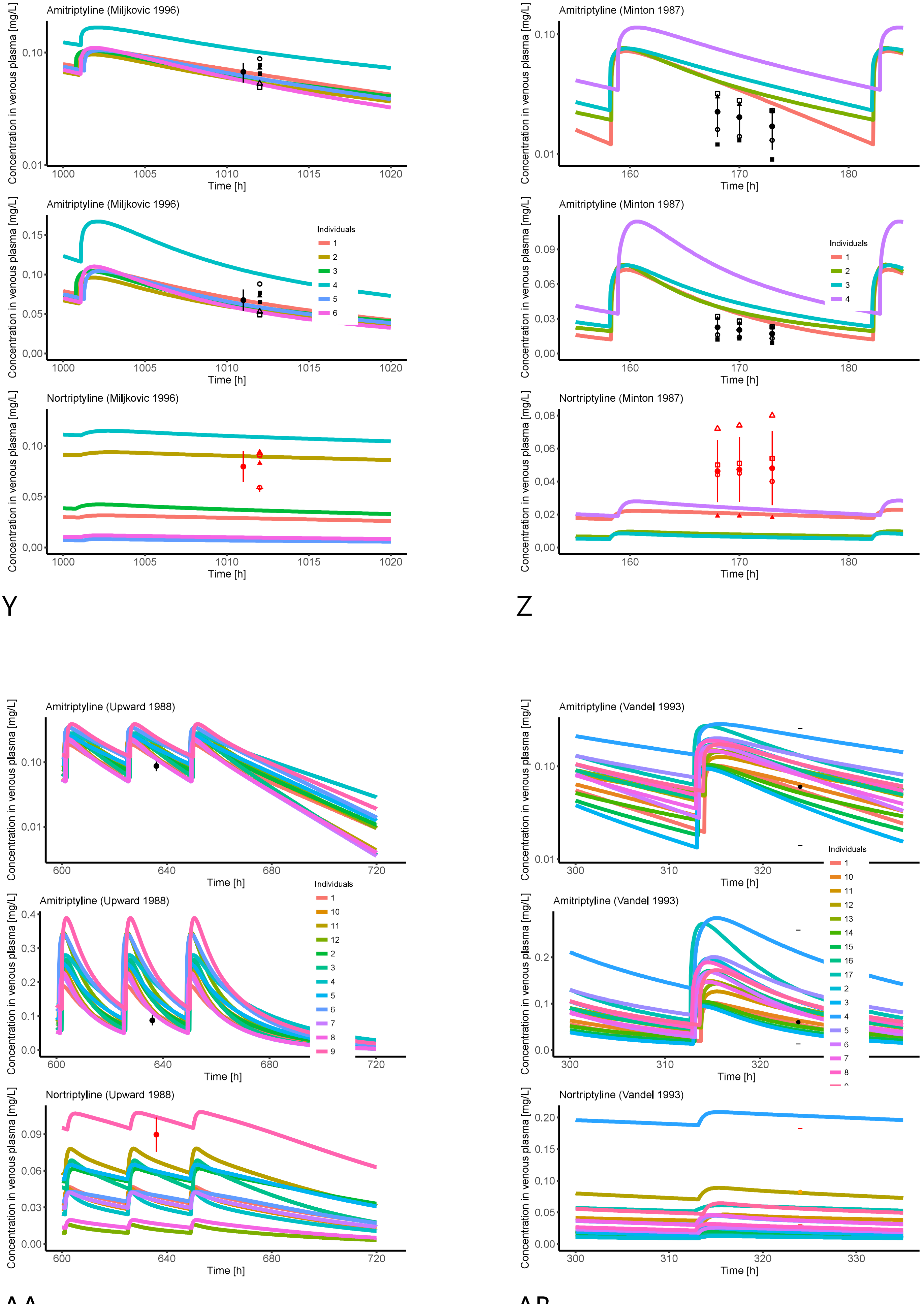

Figure 1 - Continued

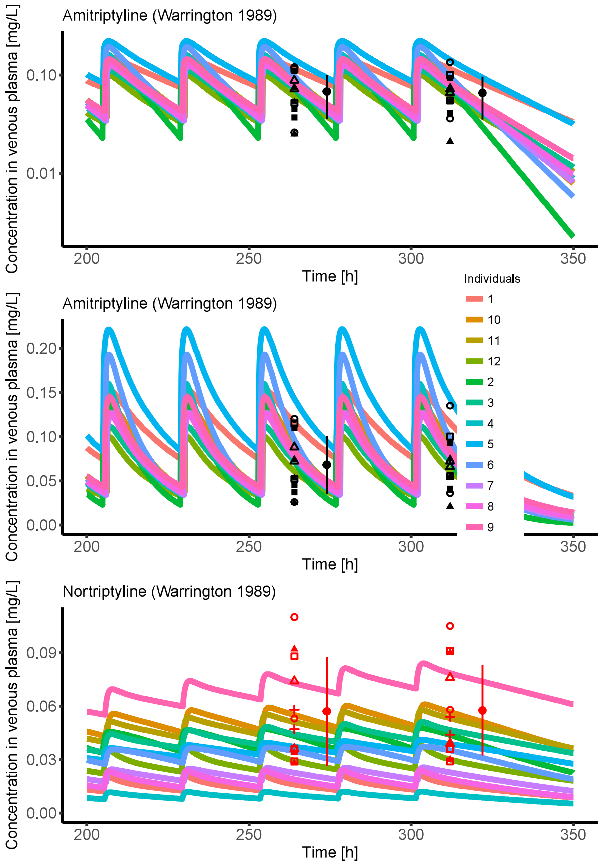

AC

Figure 2 - The PK simulations' results of cases of AT intoxication (continued on nextpage).

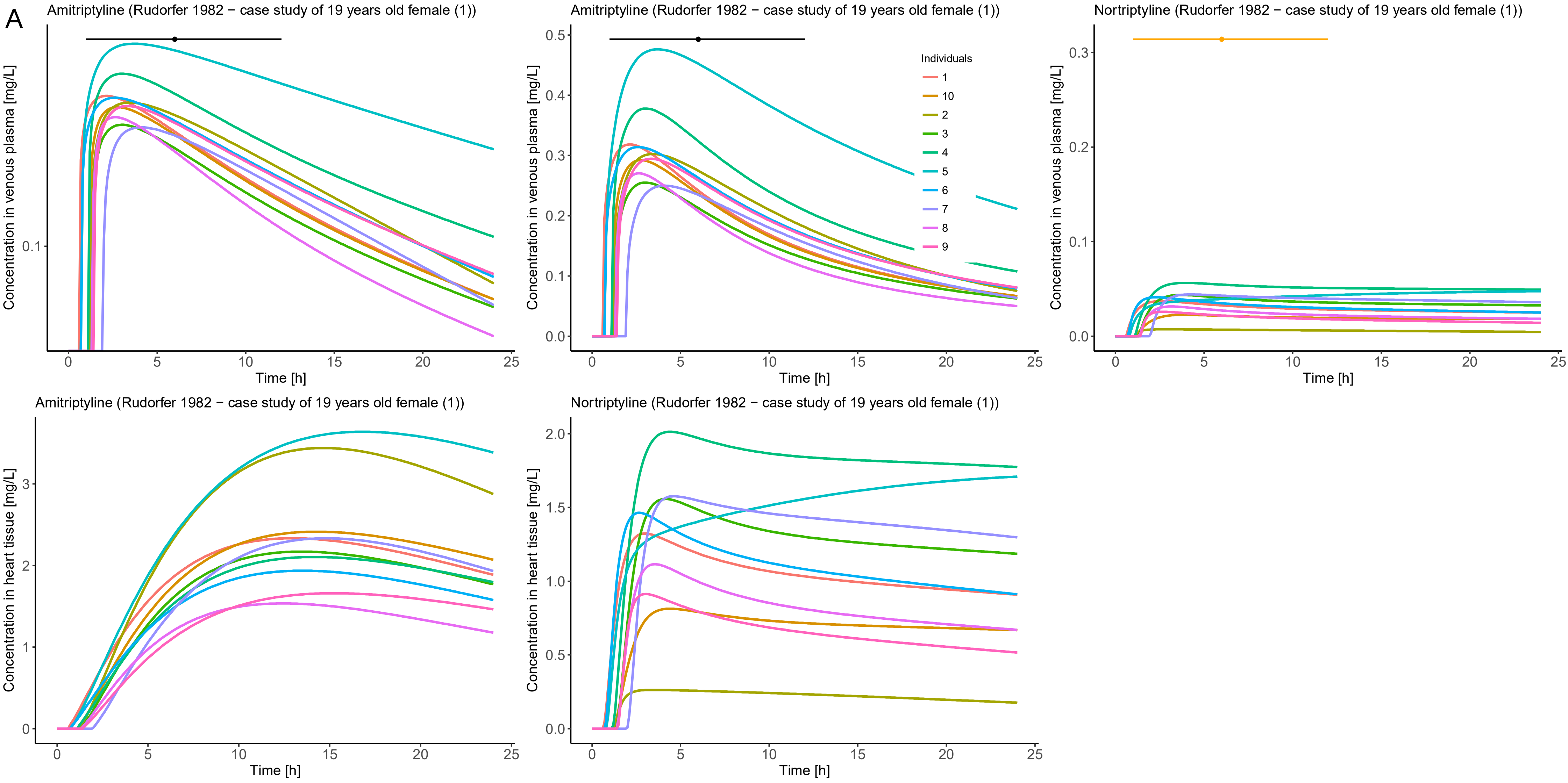

A
Amitriptyline (Rudorfer 1982 − case study of 19 years old female (1))
Concentration in venous plasma [mg/L]
Time [h]
Amitriptyline (Rudorfer 1982 − case study of 19 years old female (1))
Concentration in venous plasma [mg/L]
Time [h]
Individuals
1
10
2
3
4
5
6
7
8
9
Nortriptyline (Rudorfer 1982 − case study of 19 years old female (1))
Concentration in venous plasma [mg/L]
Time [h]
Amitriptyline (Rudorfer 1982 − case study of 19 years old female (1))
Concentration in heart tissue [mg/L]
Time [h]
Nortriptyline (Rudorfer 1982 − case study of 19 years old female (1))
Concentration in heart tissue [mg/L]
Time [h]

B

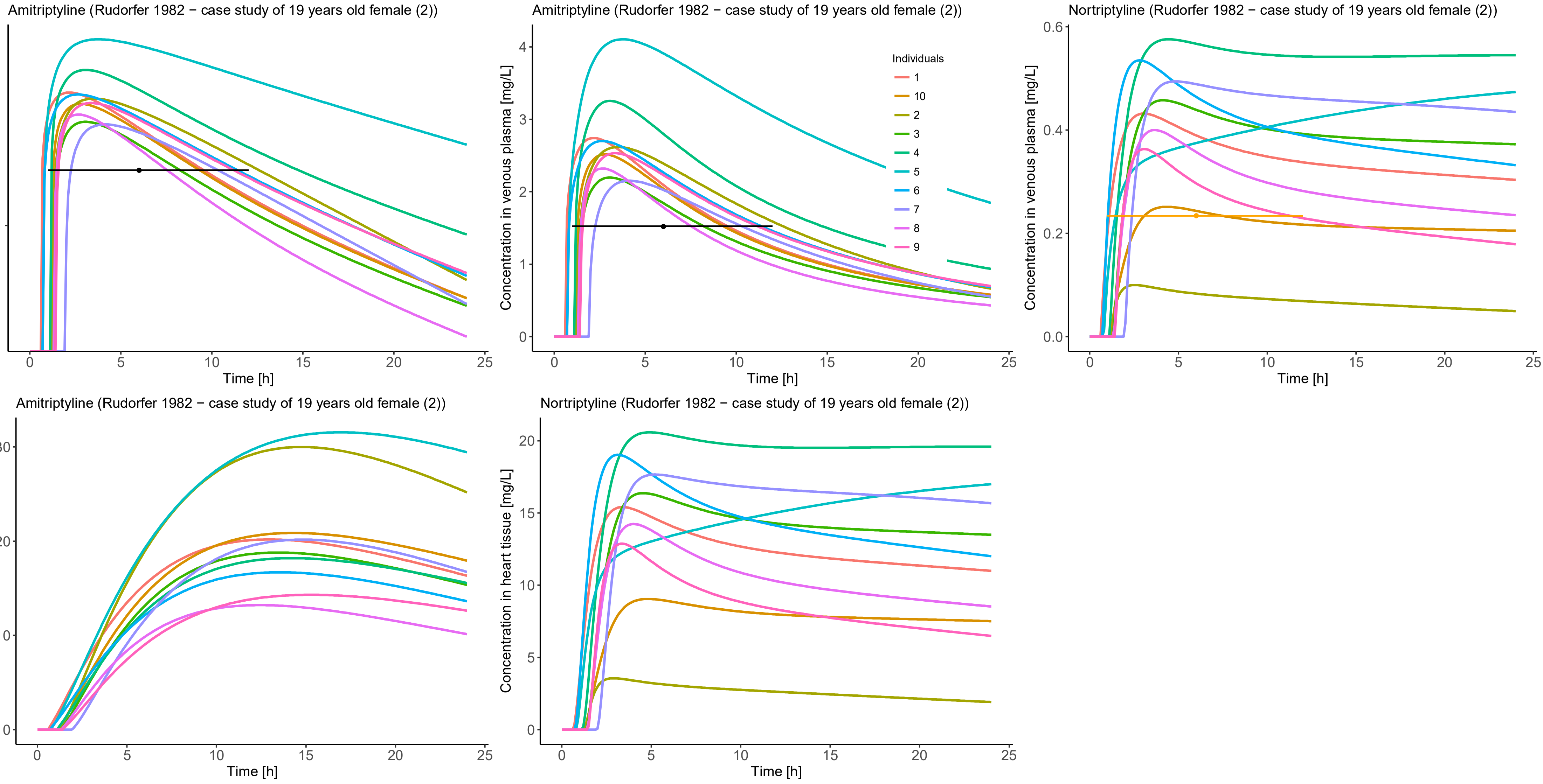

Amitriptyline (Rudorfer 1982 − case study of 19 years old female (2))
Concentration in venous plasma [mg/L]
Time [h]
Amitriptyline (Rudorfer 1982 − case study of 19 years old female (2))
Concentration in venous plasma [mg/L]
Time [h]
Individuals
1
10
2
3
4
5
6
7
8
9
Nortriptyline (Rudorfer 1982 − case study of 19 years old female (2))
Concentration in venous plasma [mg/L]
Time [h]
Amitriptyline (Rudorfer 1982 − case study of 19 years old female (2))
Concentration in heart tissue [mg/L]
Time [h]
Nortriptyline (Rudorfer 1982 − case study of 19 years old female (2))
Concentration in heart tissue [mg/L]
Time [h]

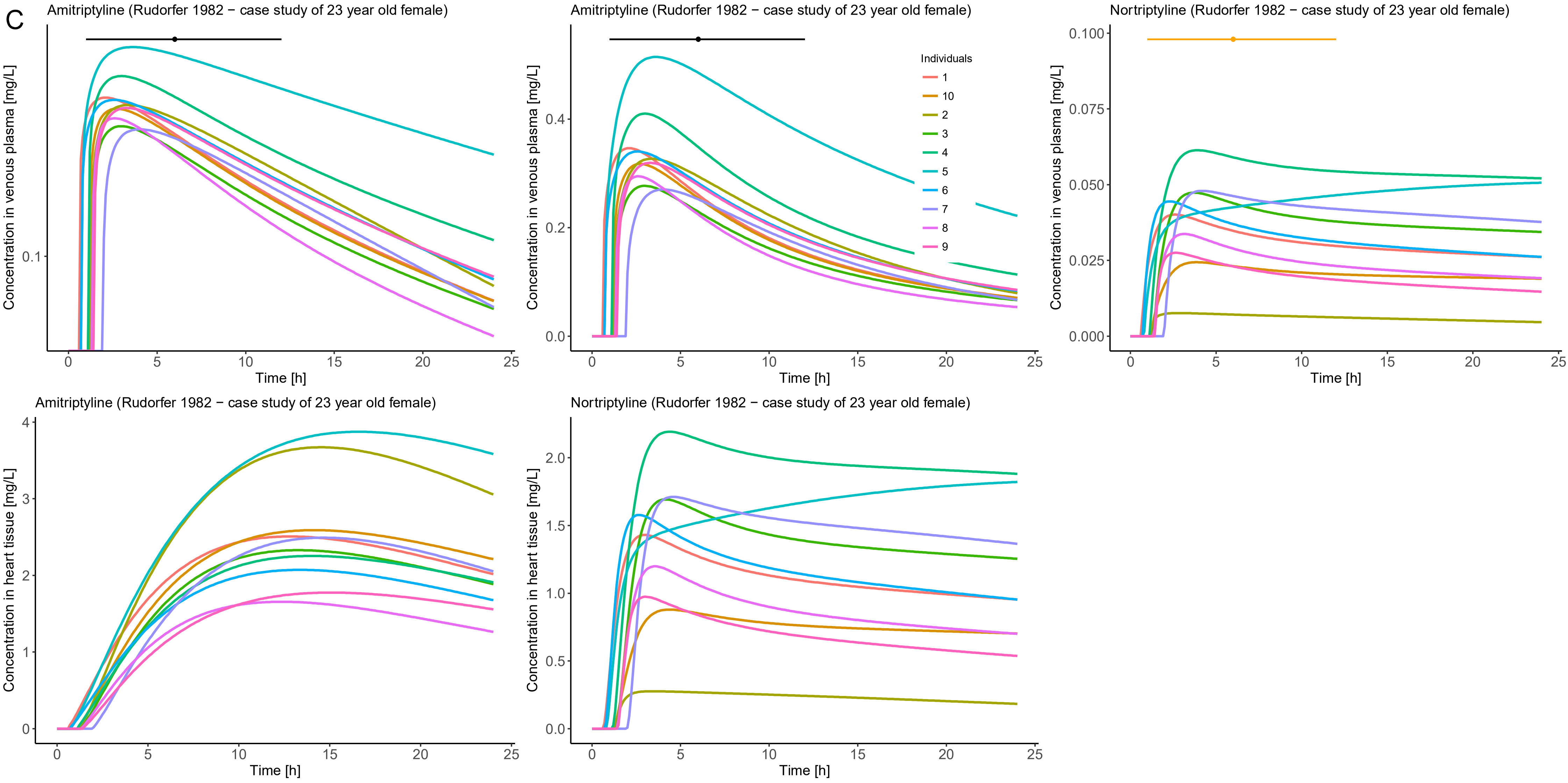
C
Amitriptyline (Rudorfer 1982 − case study of 23 year old female)
Concentration in venous plasma [mg/L]
Time [h]
Amitriptyline (Rudorfer 1982 − case study of 23 year old female)
Concentration in venous plasma [mg/L]
Time [h]
Individuals
1
10
2
3
4
5
6
7
8
9
Nortriptyline (Rudorfer 1982 − case study of 23 year old female)
Concentration in venous plasma [mg/L]
Time [h]
Amitriptyline (Rudorfer 1982 − case study of 23 year old female)
Concentration in heart tissue [mg/L]
Time [h]
Nortriptyline (Rudorfer 1982 − case study of 23 year old female)
Concentration in heart tissue [mg/L]
Time [h]

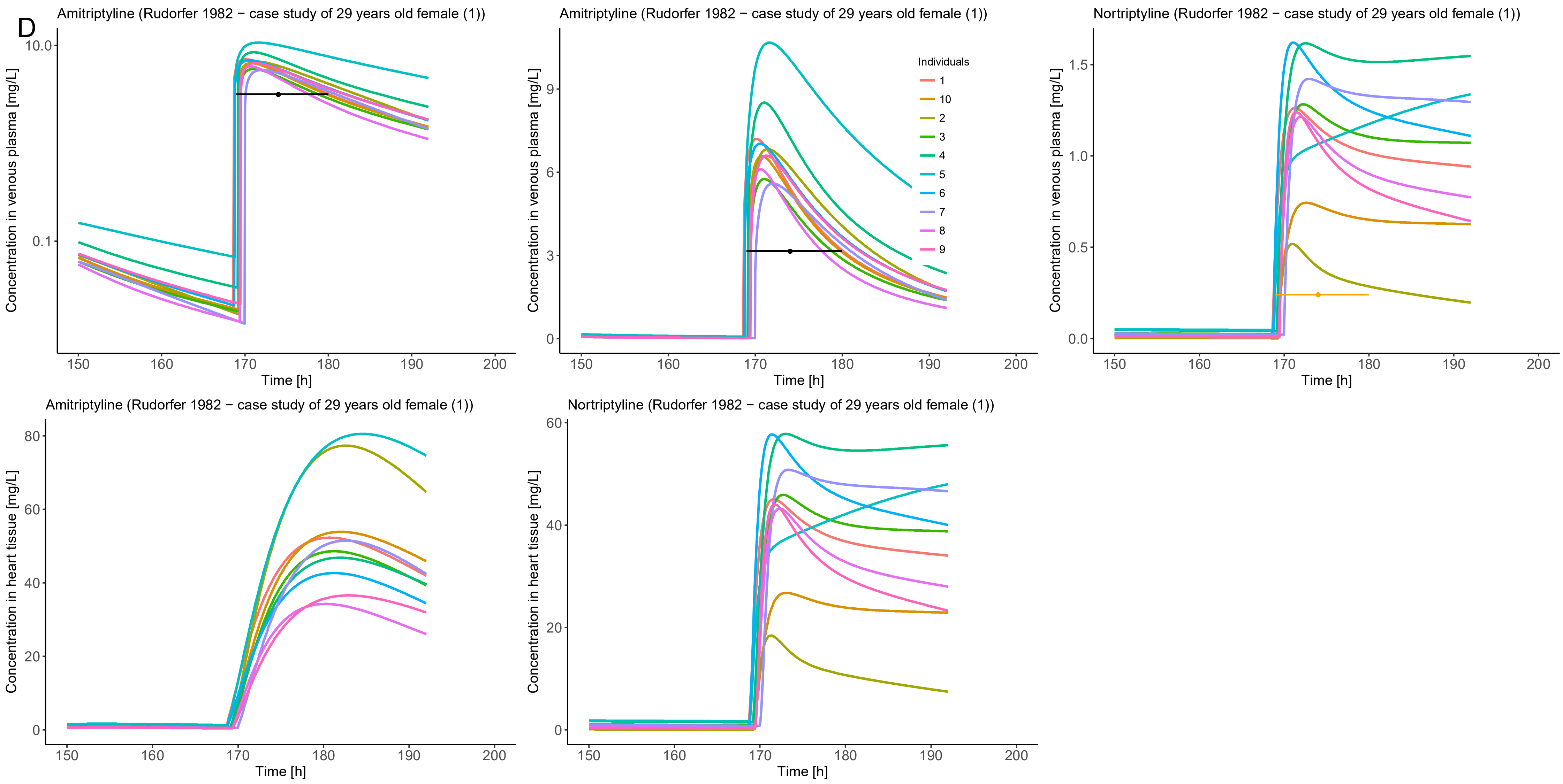
D
Amitriptyline (Rudorfer 1982 − case study of 29 years old female (1))
Concentration in venous plasma [mg/L]
Time [h]
Amitriptyline (Rudorfer 1982 − case study of 29 years old female (1))
Concentration in venous plasma [mg/L]
Time [h]
Individuals
1
10
2
3
4
5
6
7
8
9
Nortriptyline (Rudorfer 1982 − case study of 29 years old female (1))
Concentration in venous plasma [mg/L]
Time [h]
Amitriptyline (Rudorfer 1982 − case study of 29 years old female (1))
Concentration in heart tissue [mg/L]
Time [h]
Nortriptyline (Rudorfer 1982 − case study of 29 years old female (1))
Concentration in heart tissue [mg/L]
Time [h]

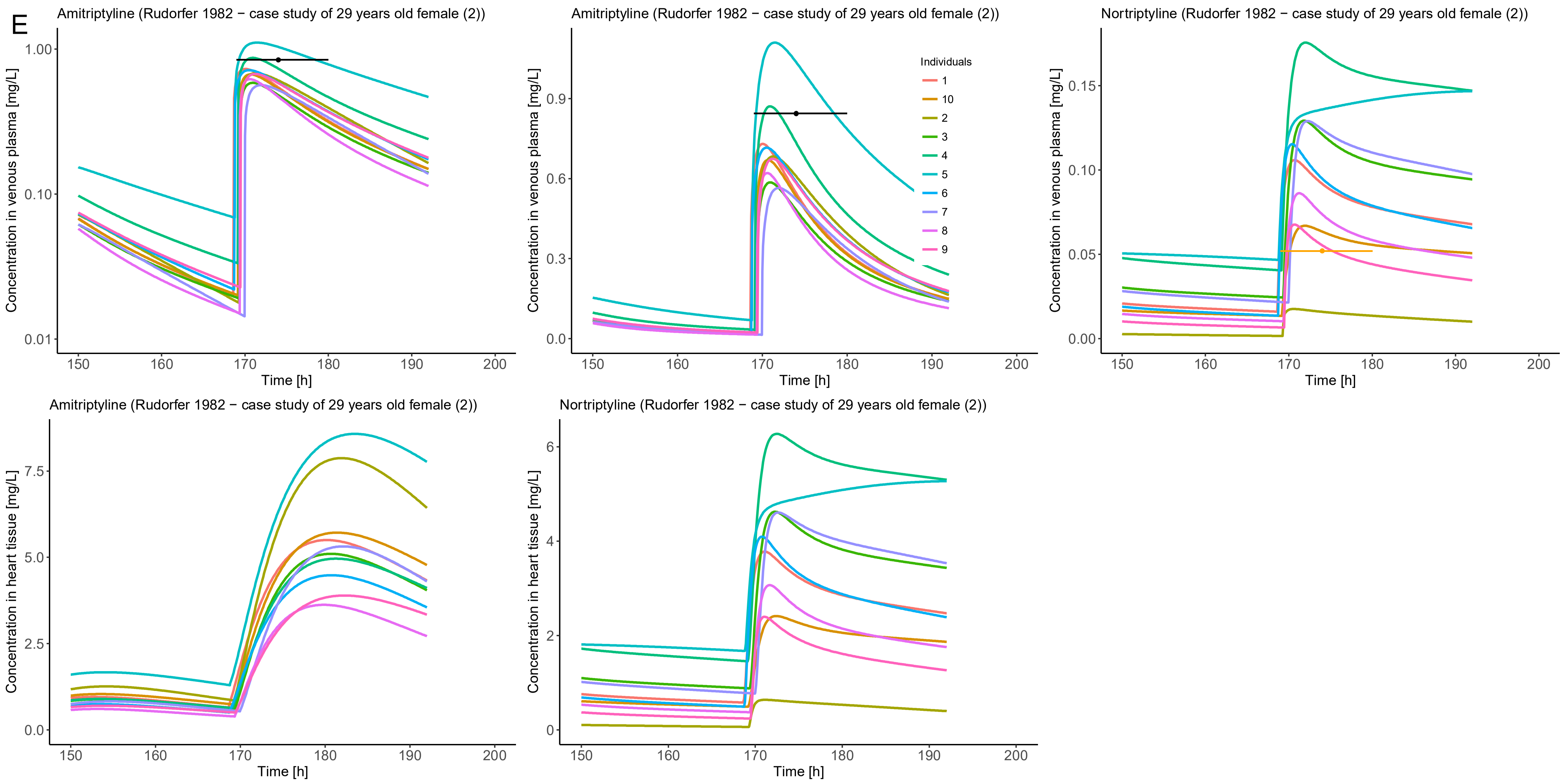
E
Amitriptyline (Rudorfer 1982 − case study of 29 years old female (2))
Concentration in venous plasma [mg/L]
1.00
0.10
0.01
Time [h]
150
160
170
180
190
200
Amitriptyline (Rudorfer 1982 − case study of 29 years old female (2))
Concentration in venous plasma [mg/L]
0.9
0.6
0.3
0.0
Individuals
1
10
2
3
4
5
6
7
8
9
Time [h]
Nortriptyline (Rudorfer 1982 − case study of 29 years old female (2))
Concentration in venous plasma [mg/L]
0.15
0.10
0.05
0.00
Time [h]
Amitriptyline (Rudorfer 1982 − case study of 29 years old female (2))
Concentration in heart tissue [mg/L]
7.5
5.0
2.5
0.0
Time [h]
Nortriptyline (Rudorfer 1982 − case study of 29 years old female (2))
Concentration in heart tissue [mg/L]
6
4
2
0
Time [h]

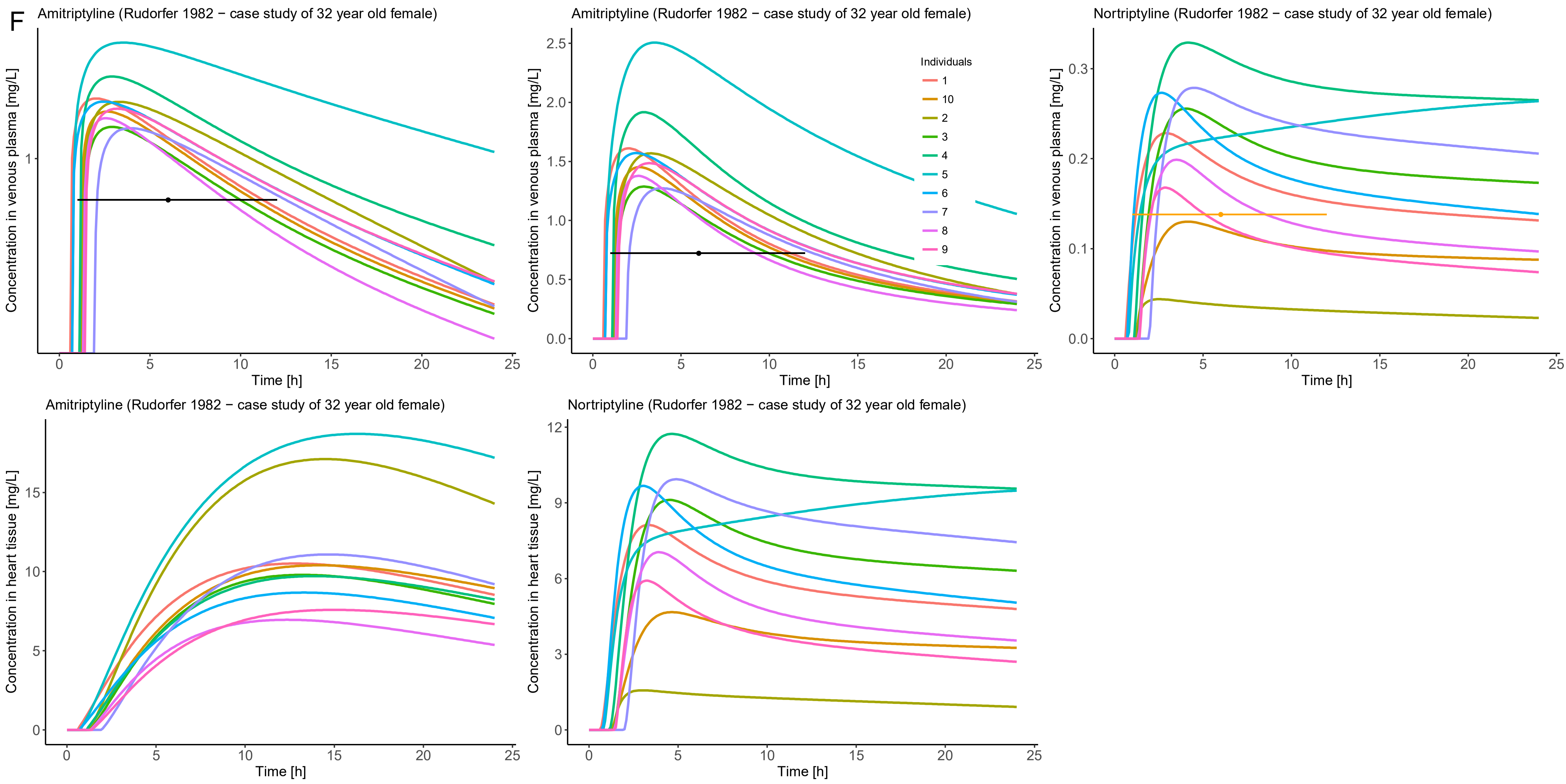
F
Amitriptyline (Rudorfer 1982 − case study of 32 year old female)
Concentration in venous plasma [mg/L]
Time [h]
Amitriptyline (Rudorfer 1982 − case study of 32 year old female)
Concentration in venous plasma [mg/L]
Time [h]
Individuals
1
10
2
3
4
5
6
7
8
9
Nortriptyline (Rudorfer 1982 − case study of 32 year old female)
Concentration in venous plasma [mg/L]
Time [h]
Amitriptyline (Rudorfer 1982 − case study of 32 year old female)
Concentration in heart tissue [mg/L]
Time [h]
Nortriptyline (Rudorfer 1982 − case study of 32 year old female)
Concentration in heart tissue [mg/L]
Time [h]

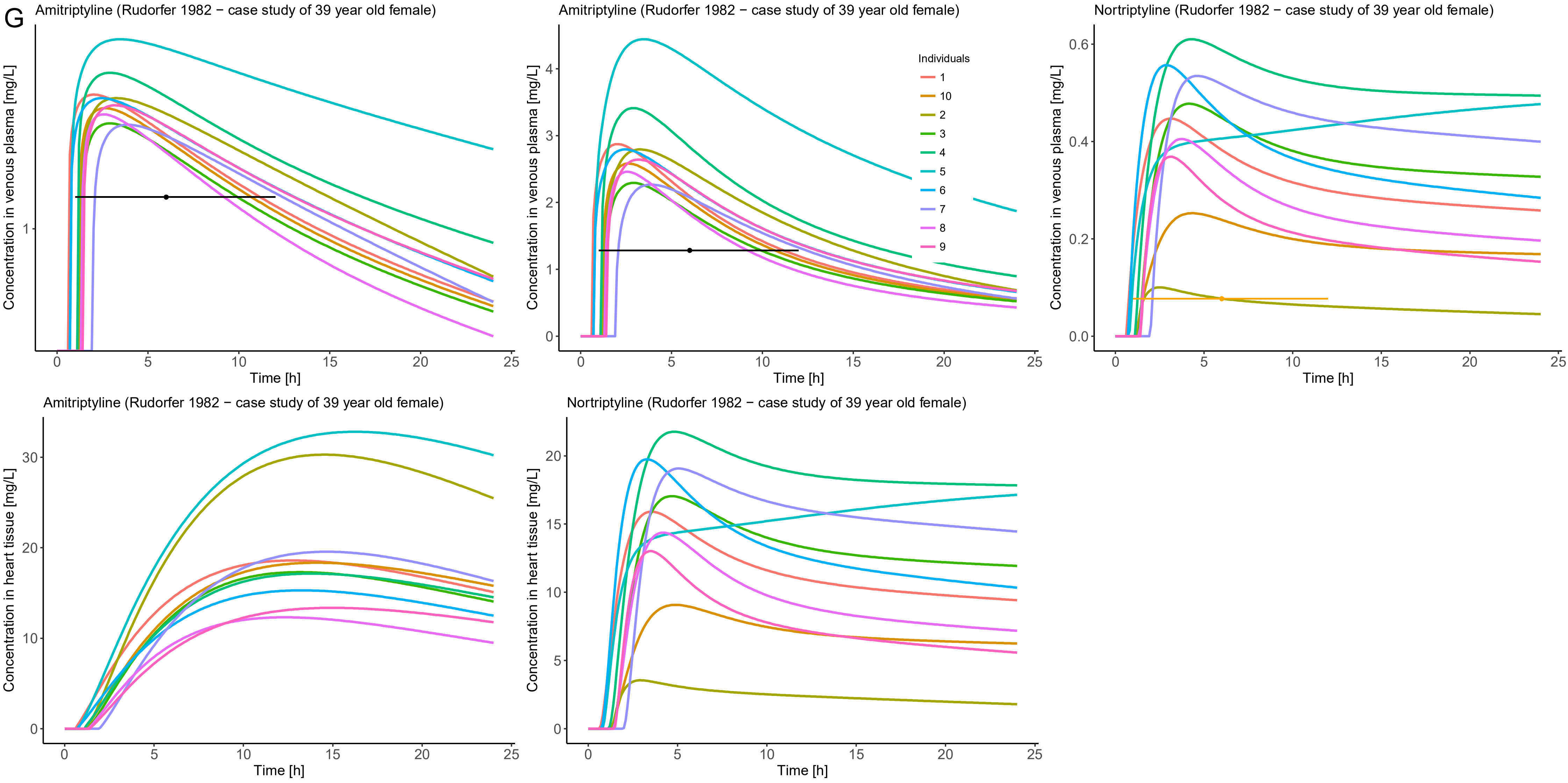
G
Amitriptyline (Rudorfer 1982 − case study of 39 year old female)
Concentration in venous plasma [mg/L]
Time [h]
Amitriptyline (Rudorfer 1982 − case study of 39 year old female)
Concentration in venous plasma [mg/L]
Time [h]
Individuals
1
10
2
3
4
5
6
7
8
9
Nortriptyline (Rudorfer 1982 − case study of 39 year old female)
Concentration in venous plasma [mg/L]
Time [h]
Amitriptyline (Rudorfer 1982 − case study of 39 year old female)
Concentration in heart tissue [mg/L]
Time [h]
Nortriptyline (Rudorfer 1982 − case study of 39 year old female)
Concentration in heart tissue [mg/L]
Time [h]

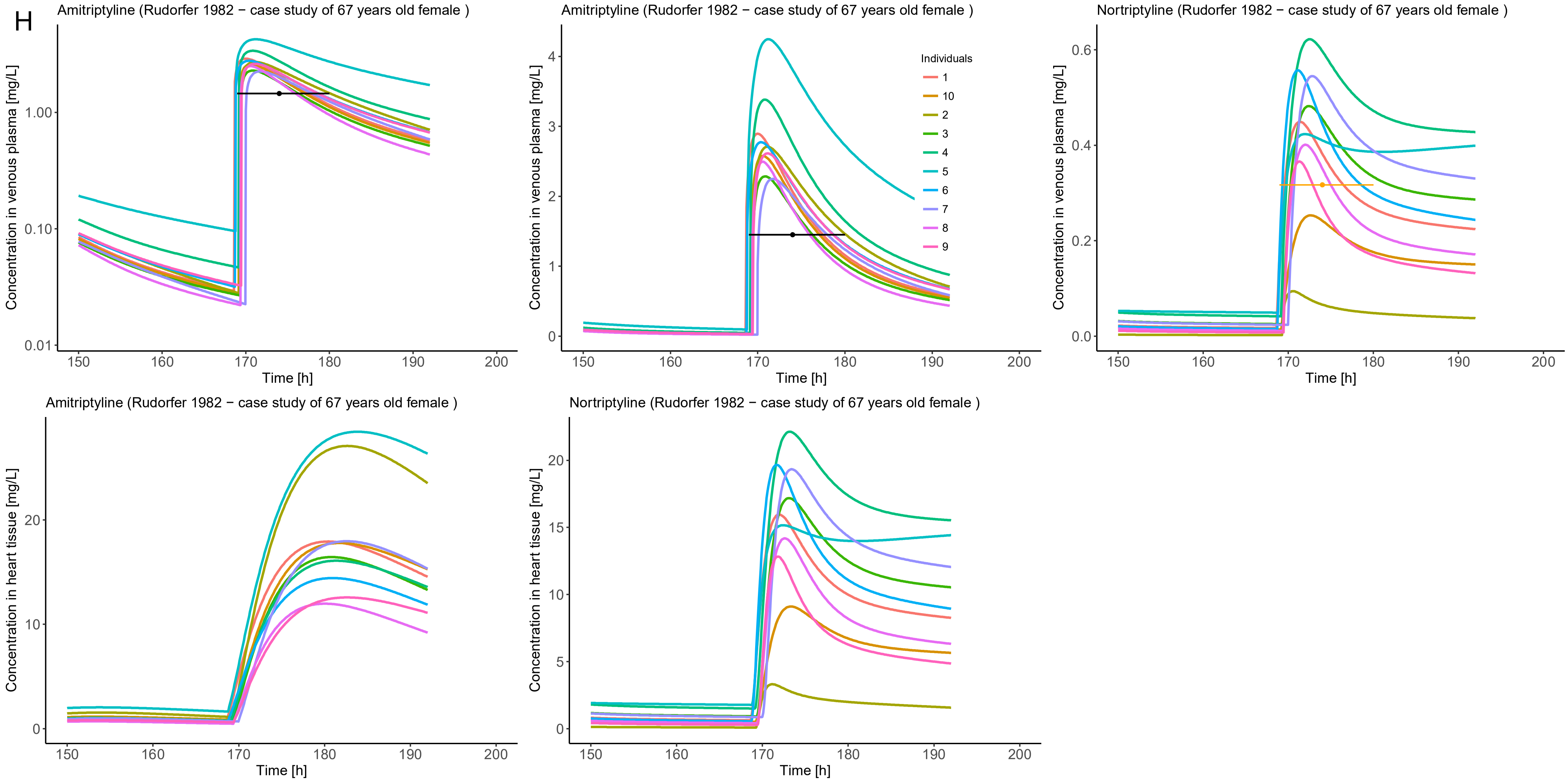
H
Amitriptyline (Rudorfer 1982 − case study of 67 years old female)
Concentration in venous plasma [mg/L]
Time [h]
Amitriptyline (Rudorfer 1982 − case study of 67 years old female)
Concentration in venous plasma [mg/L]
Time [h]
Individuals
1
10
2
3
4
5
6
7
8
9
Nortriptyline (Rudorfer 1982 − case study of 67 years old female)
Concentration in venous plasma [mg/L]
Time [h]
Amitriptyline (Rudorfer 1982 − case study of 67 years old female)
Concentration in heart tissue [mg/L]
Time [h]
Nortriptyline (Rudorfer 1982 − case study of 67 years old female)
Concentration in heart tissue [mg/L]
Time [h]

I

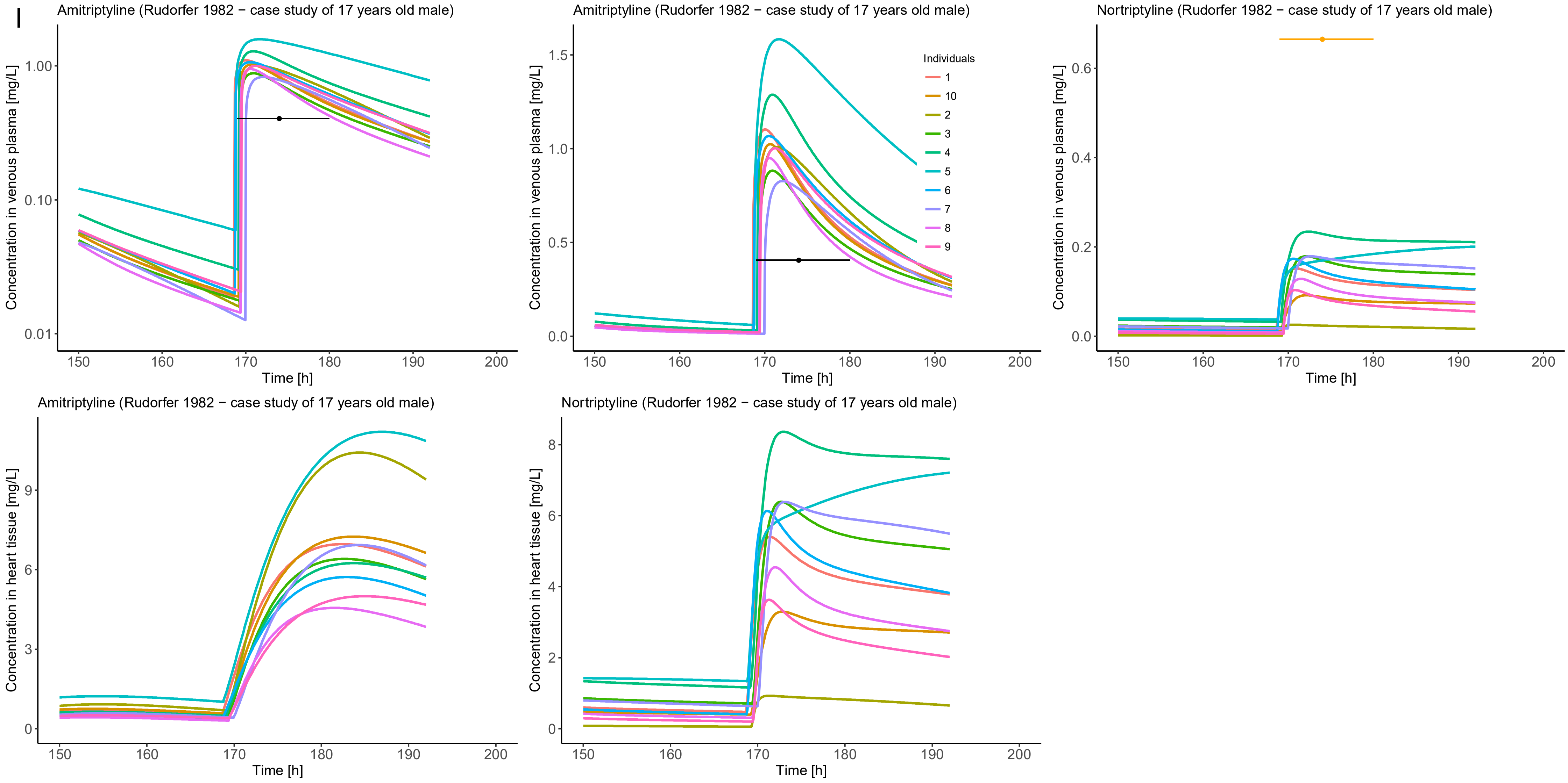

Amitriptyline (Rudorfer 1982 − case study of 17 years old male)
Concentration in venous plasma [mg/L]
1.00
0.10
0.01
Time [h]
150
160
170
180
190
200
Amitriptyline (Rudorfer 1982 − case study of 17 years old male)
Concentration in venous plasma [mg/L]
1.5
1.0
0.5
0.0
Time [h]
Individuals
1
10
2
3
4
5
6
7
8
9
Nortriptyline (Rudorfer 1982 − case study of 17 years old male)
Concentration in venous plasma [mg/L]
0.6
0.4
0.2
0.0
Time [h]
Amitriptyline (Rudorfer 1982 − case study of 17 years old male)
Concentration in heart tissue [mg/L]
9
6
3
0
Time [h]
Nortriptyline (Rudorfer 1982 − case study of 17 years old male)
Concentration in heart tissue [mg/L]
8
6
4
2
0
Time [h]

J

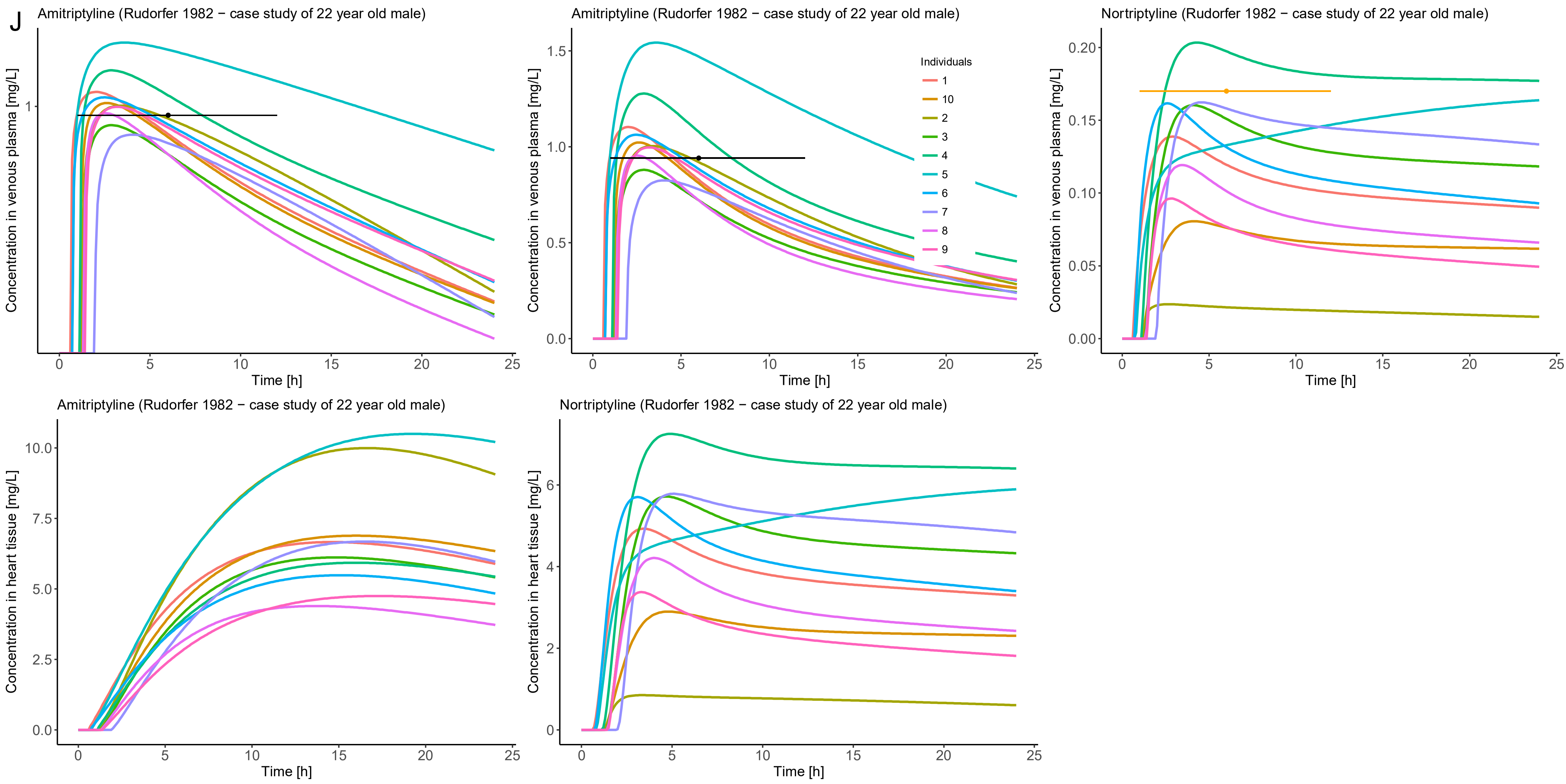

Amitriptyline (Rudorfer 1982 − case study of 22 year old male)
Concentration in venous plasma [mg/L]
Time [h]
Amitriptyline (Rudorfer 1982 − case study of 22 year old male)
Concentration in venous plasma [mg/L]
Time [h]
Individuals
1
10
2
3
4
5
6
7
8
9
Nortriptyline (Rudorfer 1982 − case study of 22 year old male)
Concentration in venous plasma [mg/L]
Time [h]
Amitriptyline (Rudorfer 1982 − case study of 22 year old male)
Concentration in heart tissue [mg/L]
Time [h]
Nortriptyline (Rudorfer 1982 − case study of 22 year old male)
Concentration in heart tissue [mg/L]
Time [h]

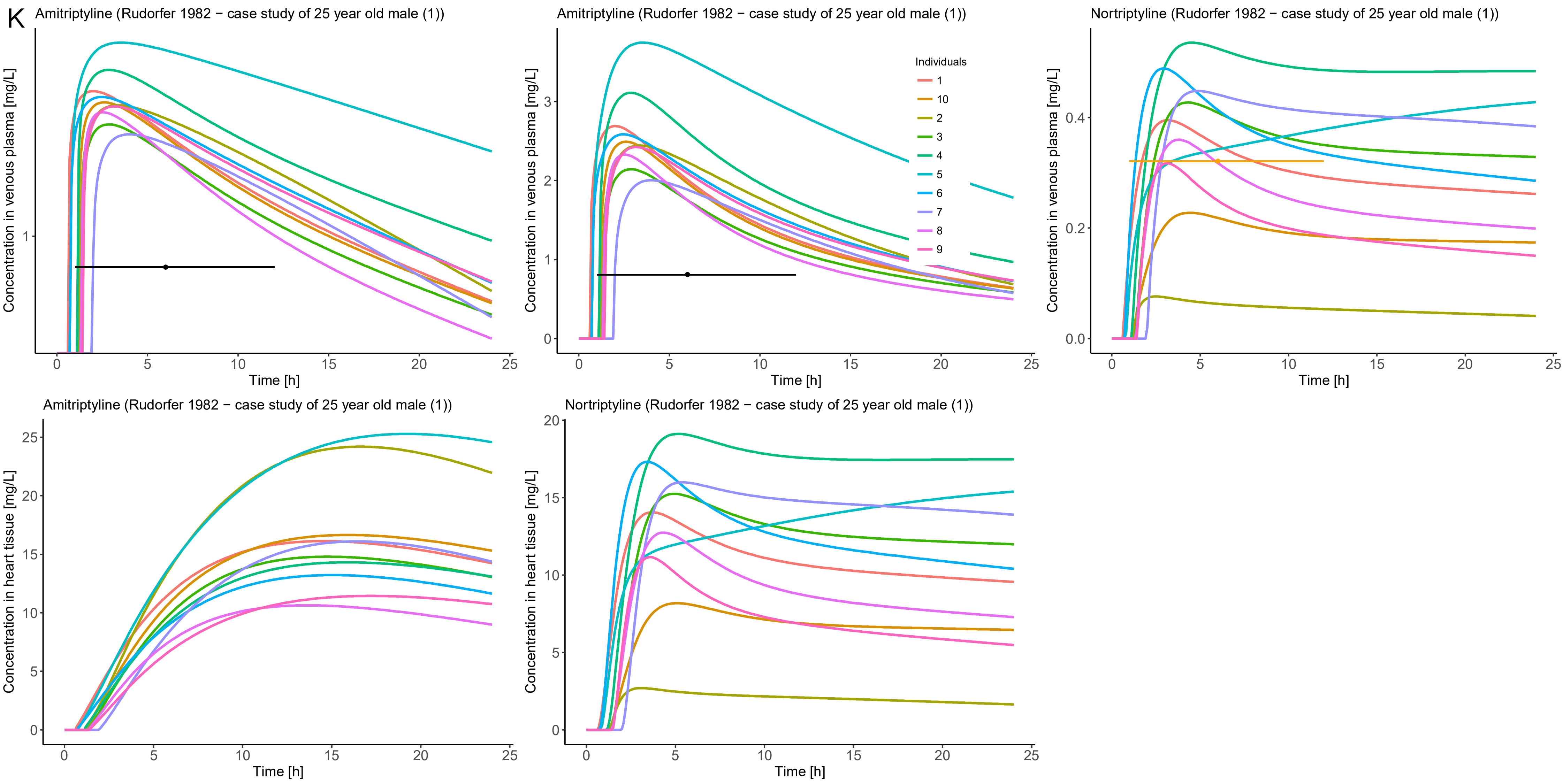
K
Amitriptyline (Rudorfer 1982 − case study of 25 year old male (1))
Concentration in venous plasma [mg/L]
Time [h]
Amitriptyline (Rudorfer 1982 − case study of 25 year old male (1))
Concentration in venous plasma [mg/L]
Time [h]
Individuals
1
10
2
3
4
5
6
7
8
9
Nortriptyline (Rudorfer 1982 − case study of 25 year old male (1))
Concentration in venous plasma [mg/L]
Time [h]
Amitriptyline (Rudorfer 1982 − case study of 25 year old male (1))
Concentration in heart tissue [mg/L]
Time [h]
Nortriptyline (Rudorfer 1982 − case study of 25 year old male (1))
Concentration in heart tissue [mg/L]
Time [h]

L

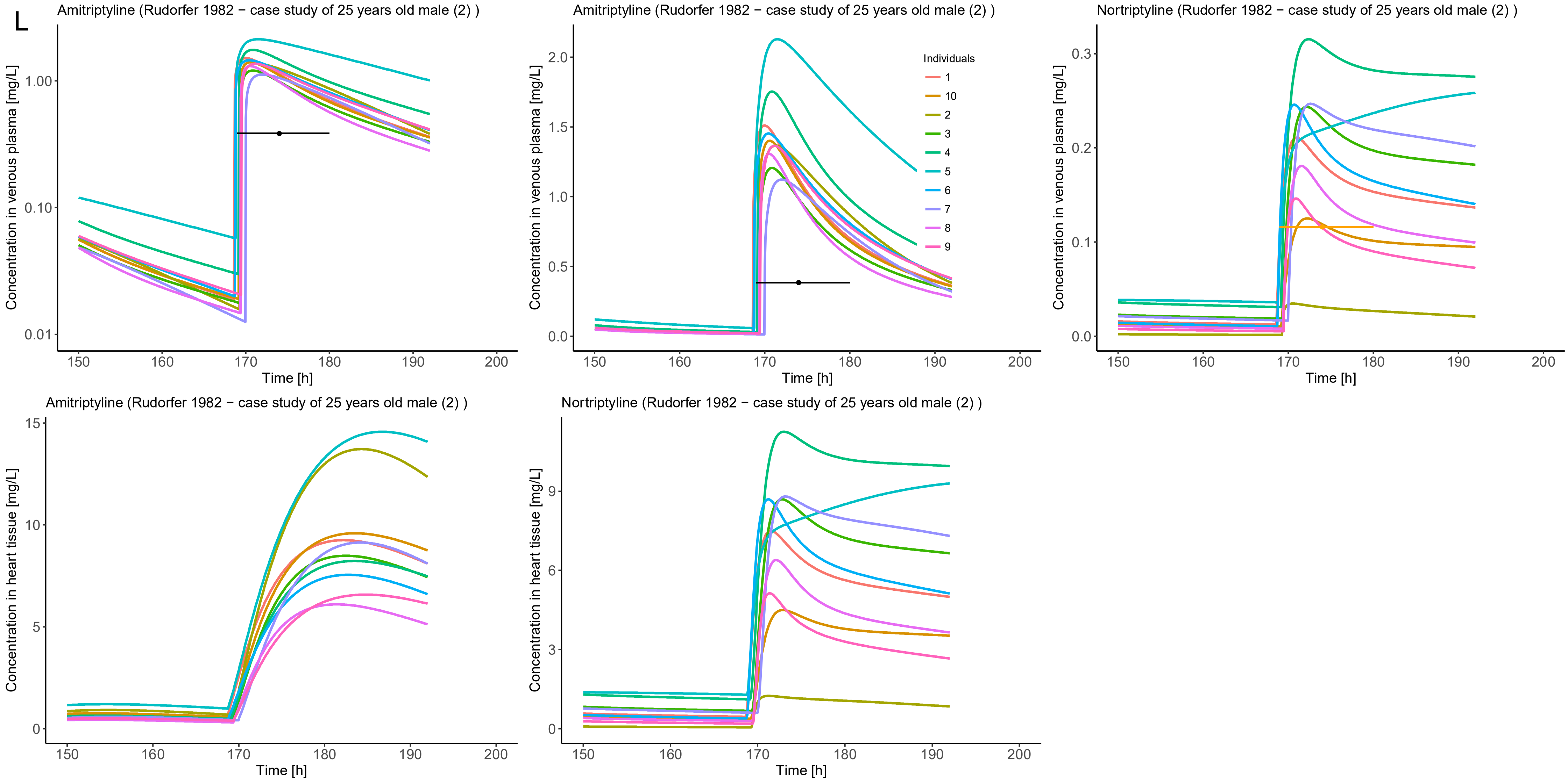
Amitriptyline (Rudorfer 1982 − case study of 25 years old male (2))
Concentration in venous plasma [mg/L]
0.01
0.10
1.00
Time [h]
150
160
170
180
190
200
Amitriptyline (Rudorfer 1982 − case study of 25 years old male (2))
Concentration in venous plasma [mg/L]
0.0
0.5
1.0
1.5
2.0
Individuals
1
10
2
3
4
5
6
7
8
9
Time [h]
Nortriptyline (Rudorfer 1982 − case study of 25 years old male (2))
Concentration in venous plasma [mg/L]
0.0
0.1
0.2
0.3
Time [h]
Amitriptyline (Rudorfer 1982 − case study of 25 years old male (2))
Concentration in heart tissue [mg/L]
0
5
10
15
Time [h]
Nortriptyline (Rudorfer 1982 − case study of 25 years old male (2))
Concentration in heart tissue [mg/L]
0
3
6
9
Time [h]

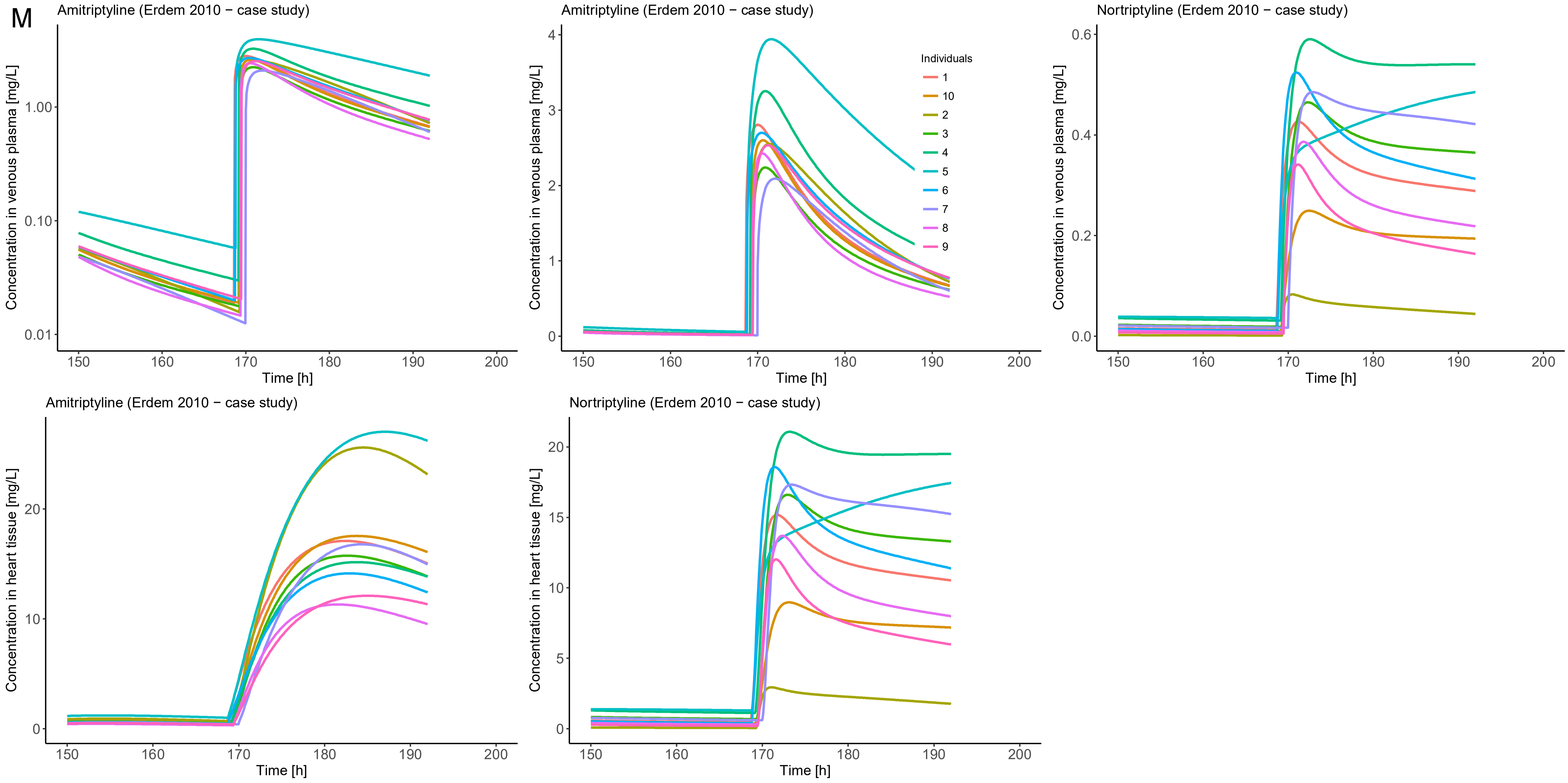
M
Amitriptyline (Erdem 2010 − case study)
Concentration in venous plasma [mg/L]
0.01
0.10
1.00
Time [h]
150
160
170
180
190
200
Amitriptyline (Erdem 2010 − case study)
Concentration in venous plasma [mg/L]
0
1
2
3
4
Time [h]
Individuals
1
10
2
3
4
5
6
7
8
9
Nortriptyline (Erdem 2010 − case study)
Concentration in venous plasma [mg/L]
0.0
0.2
0.4
0.6
Time [h]
Amitriptyline (Erdem 2010 − case study)
Concentration in heart tissue [mg/L]
0
10
20
Time [h]
Nortriptyline (Erdem 2010 − case study)
Concentration in heart tissue [mg/L]
0
5
10
15
20
Time [h]

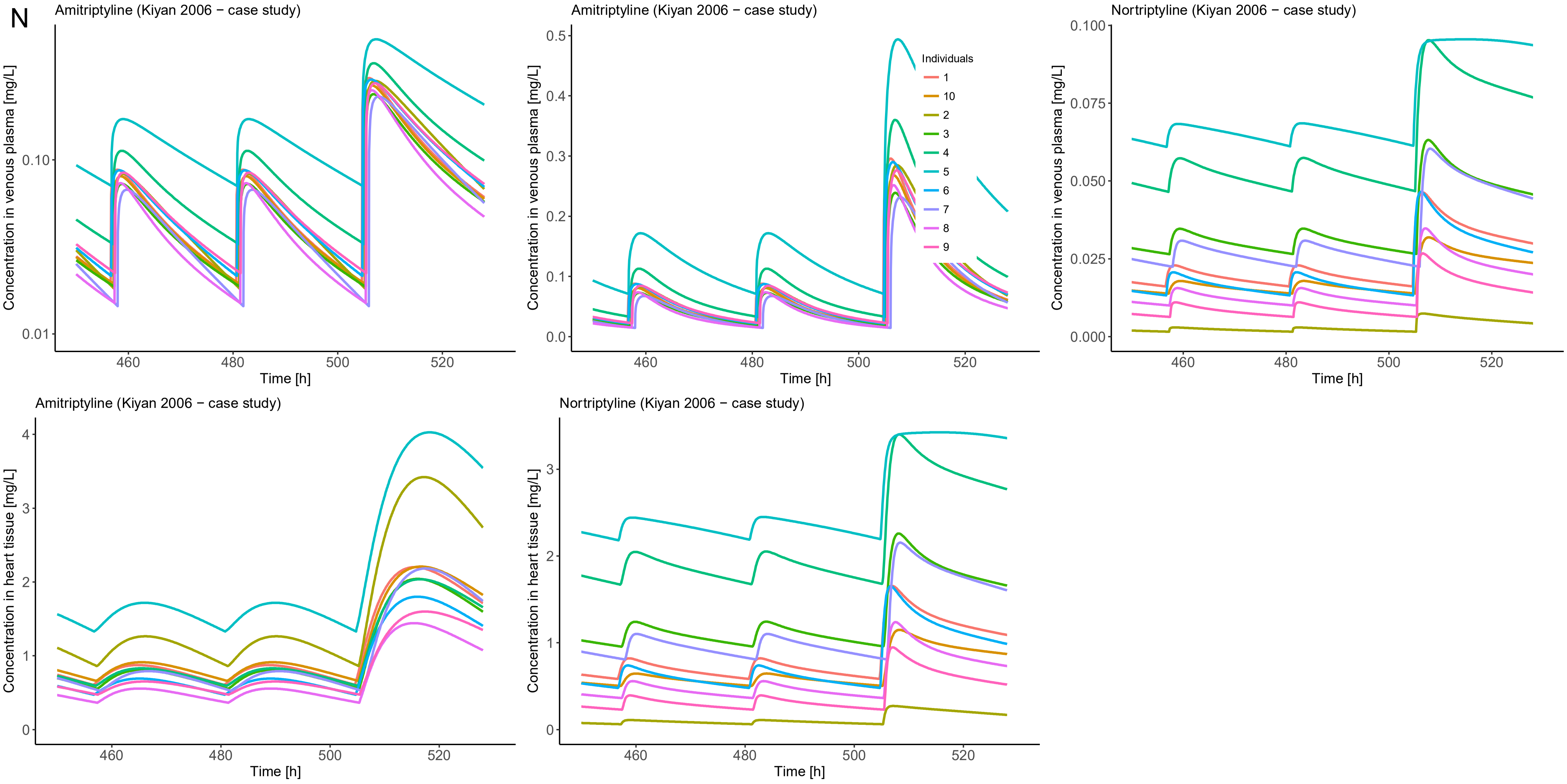
N
Amitriptyline (Kiyan 2006 − case study)
Concentration in venous plasma [mg/L]
0.10
0.01
460
480
500
520
Time [h]
Amitriptyline (Kiyan 2006 − case study)
Concentration in venous plasma [mg/L]
0.5
0.4
0.3
0.2
0.1
0.0
Individuals
1
10
2
3
4
5
6
7
8
9
Time [h]
Nortriptyline (Kiyan 2006 − case study)
Concentration in venous plasma [mg/L]
0.100
0.075
0.050
0.025
0.000
Time [h]
Amitriptyline (Kiyan 2006 − case study)
Concentration in heart tissue [mg/L]
4
3
2
1
0
Time [h]
Nortriptyline (Kiyan 2006 − case study)
Concentration in heart tissue [mg/L]
3
2
1
0
Time [h]

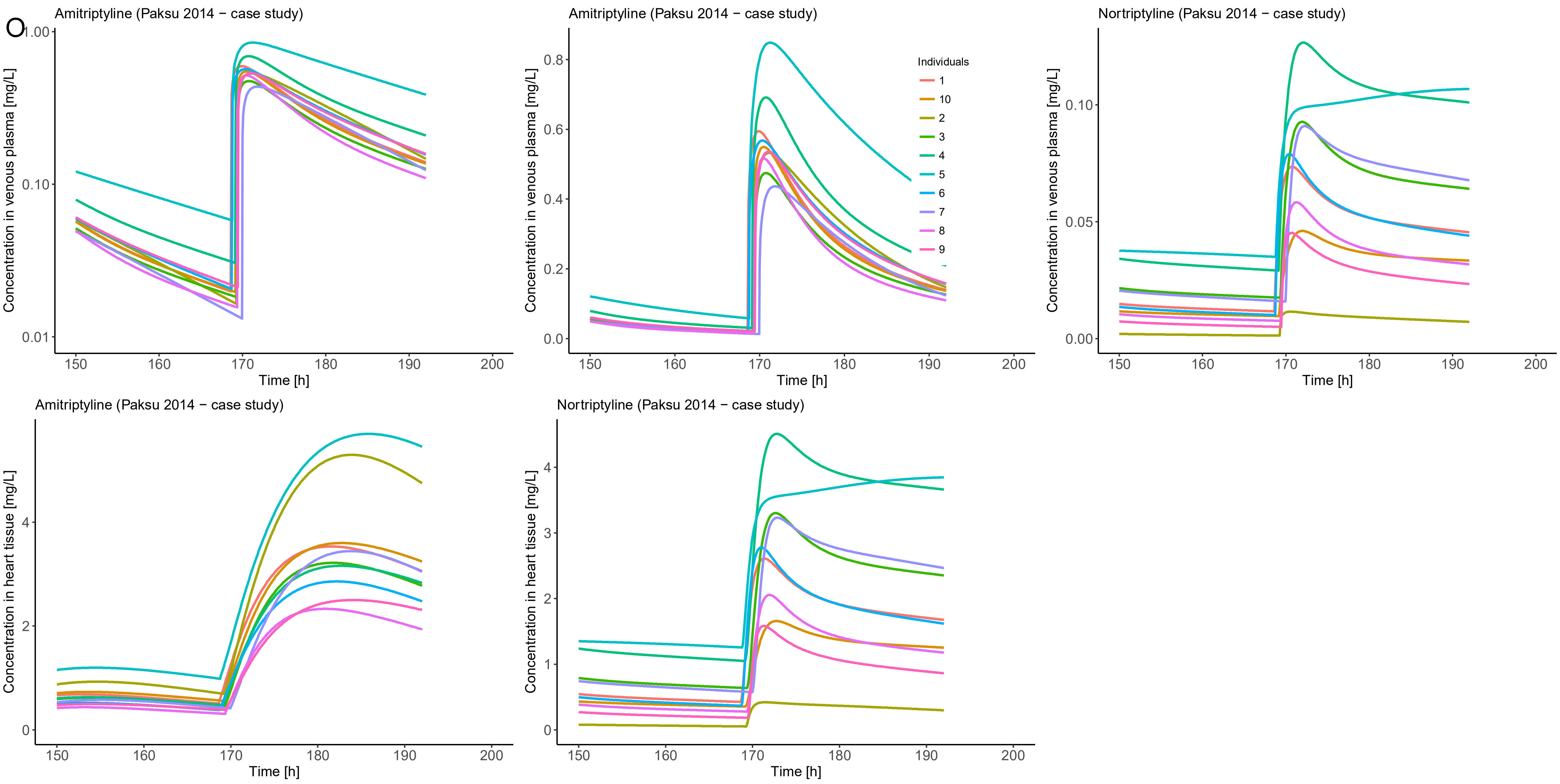
O
Amitriptyline (Paksu 2014 − case study)
Concentration in venous plasma [mg/L]
1.00
0.10
0.01
150
160
170
180
190
200
Time [h]
Amitriptyline (Paksu 2014 − case study)
Concentration in venous plasma [mg/L]
0.8
0.6
0.4
0.2
0.0
Individuals
1
10
2
3
4
5
6
7
8
9
Time [h]
Nortriptyline (Paksu 2014 − case study)
Concentration in venous plasma [mg/L]
0.10
0.05
0.00
Time [h]
Amitriptyline (Paksu 2014 − case study)
Concentration in heart tissue [mg/L]
4
2
0
Time [h]
Nortriptyline (Paksu 2014 − case study)
Concentration in heart tissue [mg/L]
4
3
2
1
0
Time [h]

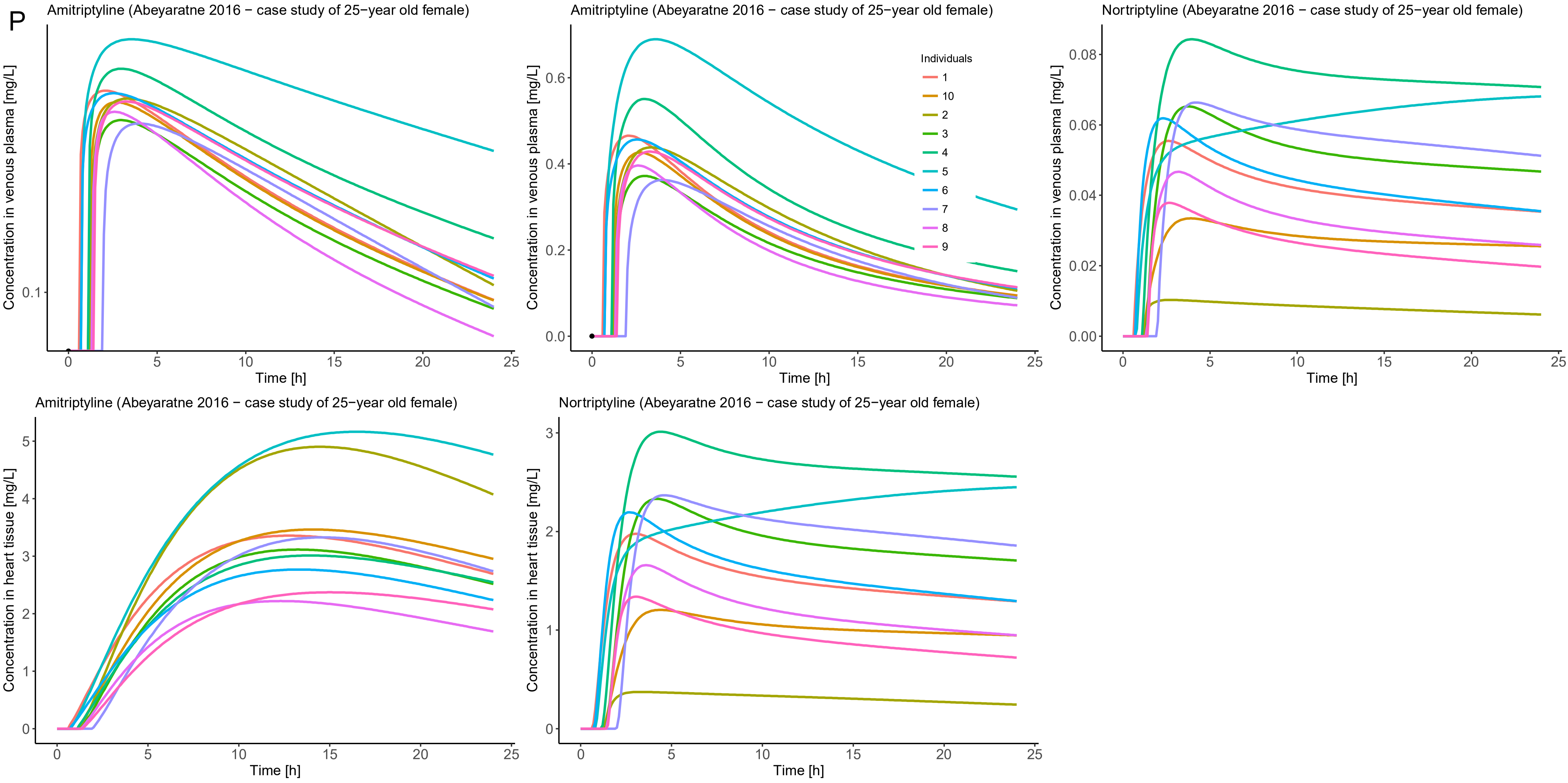

P
Amitriptyline (Abeyaratne 2016 − case study of 25−year old female)
Concentration in venous plasma [mg/L]
Time [h]
Amitriptyline (Abeyaratne 2016 − case study of 25−year old female)
Concentration in venous plasma [mg/L]
Time [h]
Individuals
1
10
2
3
4
5
6
7
8
9
Nortriptyline (Abeyaratne 2016 − case study of 25−year old female)
Concentration in venous plasma [mg/L]
Time [h]
Amitriptyline (Abeyaratne 2016 − case study of 25−year old female)
Concentration in heart tissue [mg/L]
Time [h]
Nortriptyline (Abeyaratne 2016 − case study of 25−year old female)
Concentration in heart tissue [mg/L]
Time [h]

Figure 3 - The simulated QT or QTc for each of the cases in 10 virtual patients (1 - 10) along with clinical observations (obs). The triangle means virtual arrhythmia occurrence.

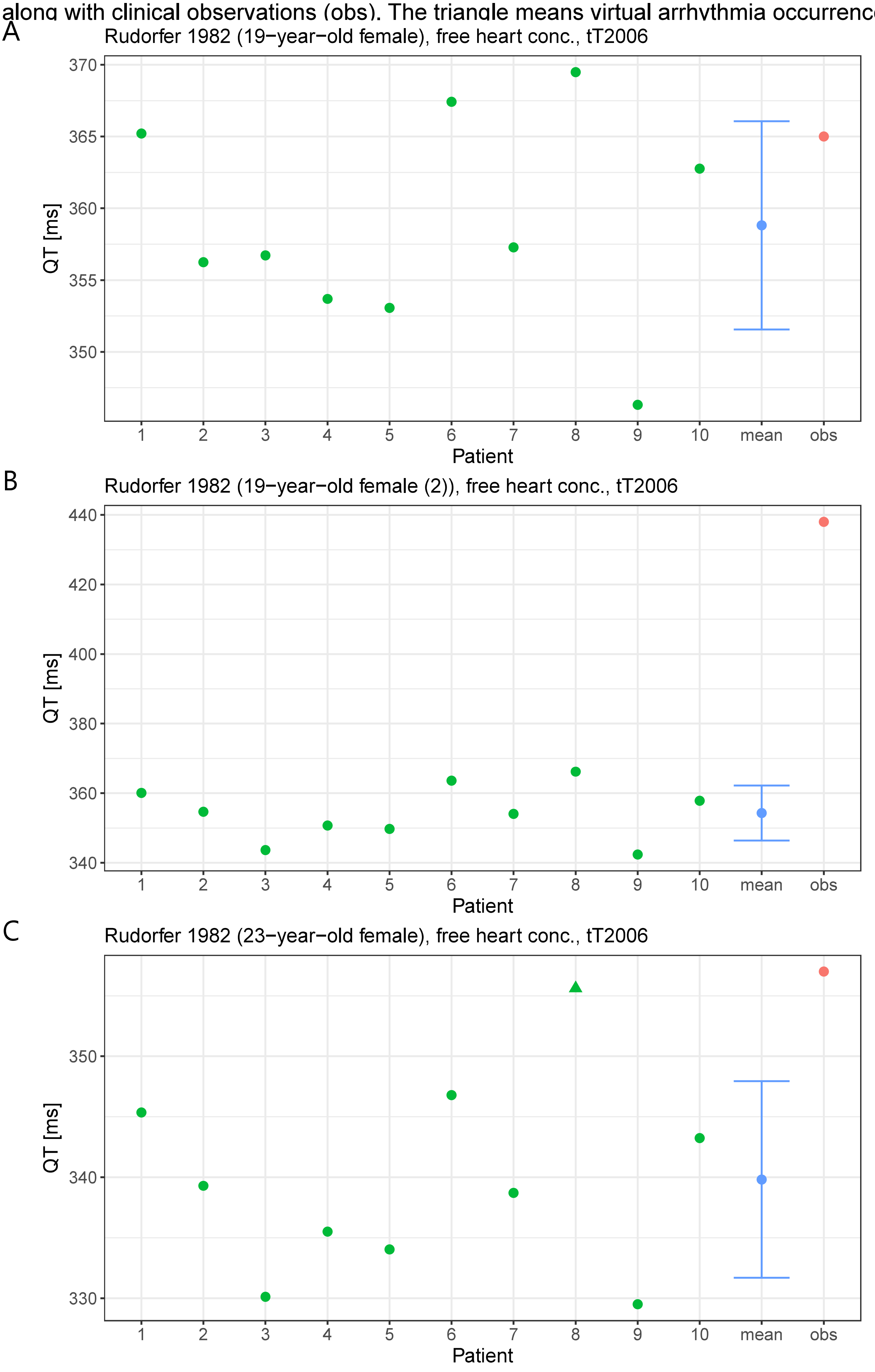

Figure 3 - Continued

D

Rudorfer 1982 (29−year−old female (1)), free heart conc., tT2006

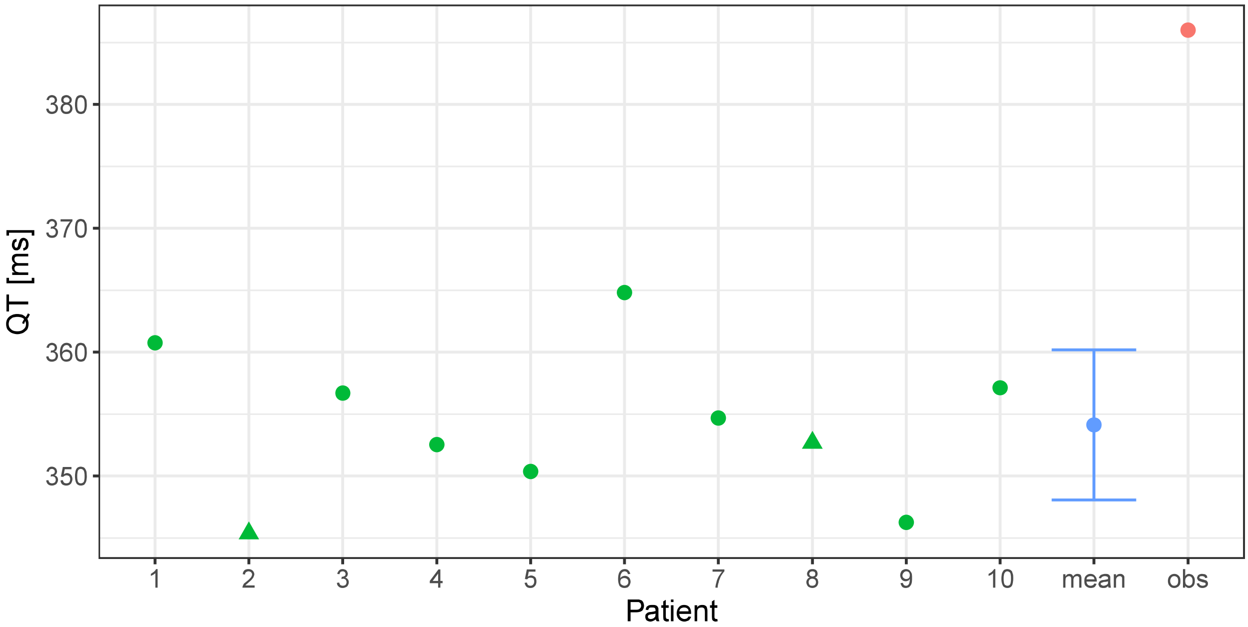

E

Rudorfer 1982 (29−year−old female (2)), free heart conc., tT2006

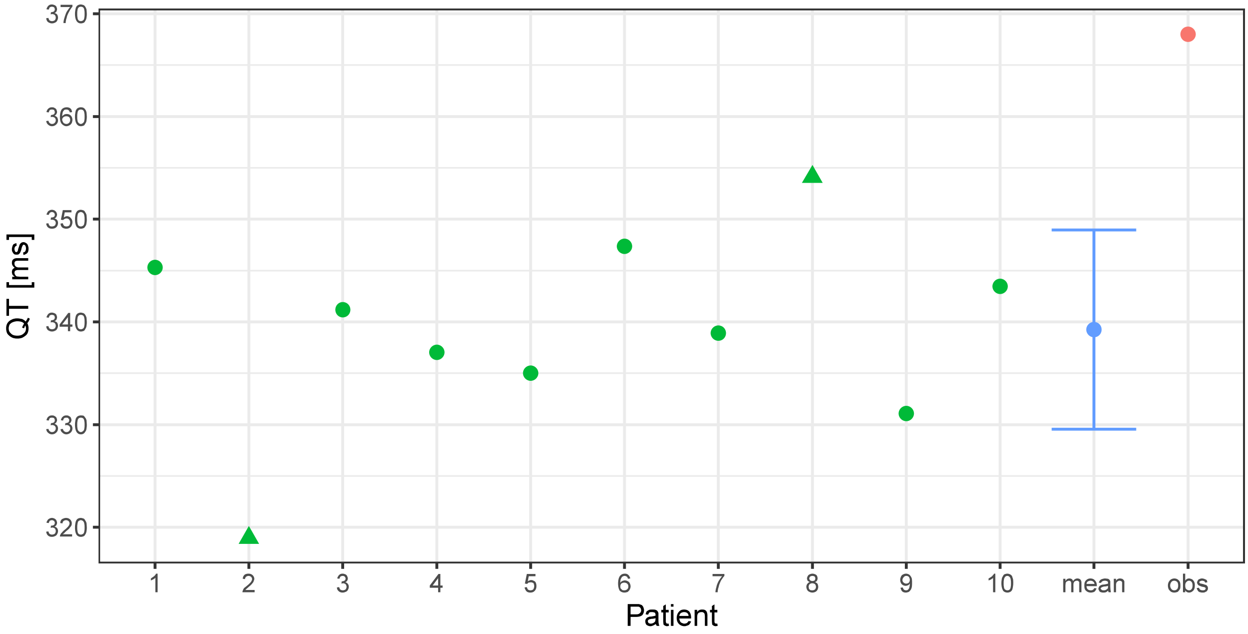

F

Rudorfer 1982 (32−year−old female), free heart conc., tT2006

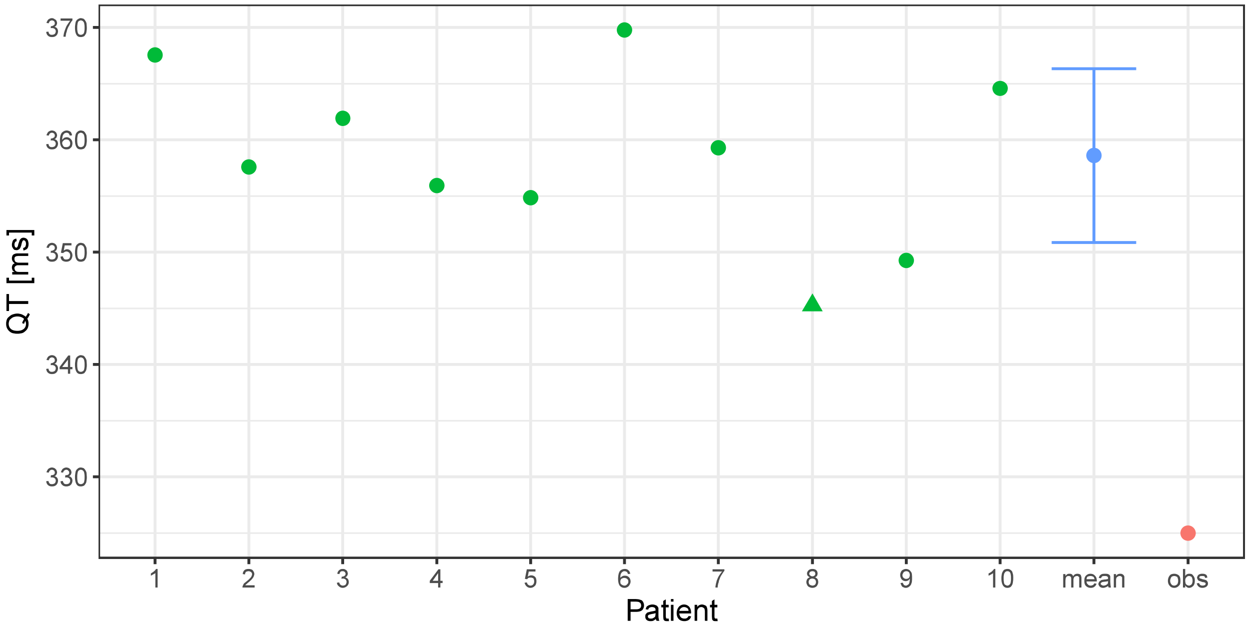

Figure 3 - Continued

G

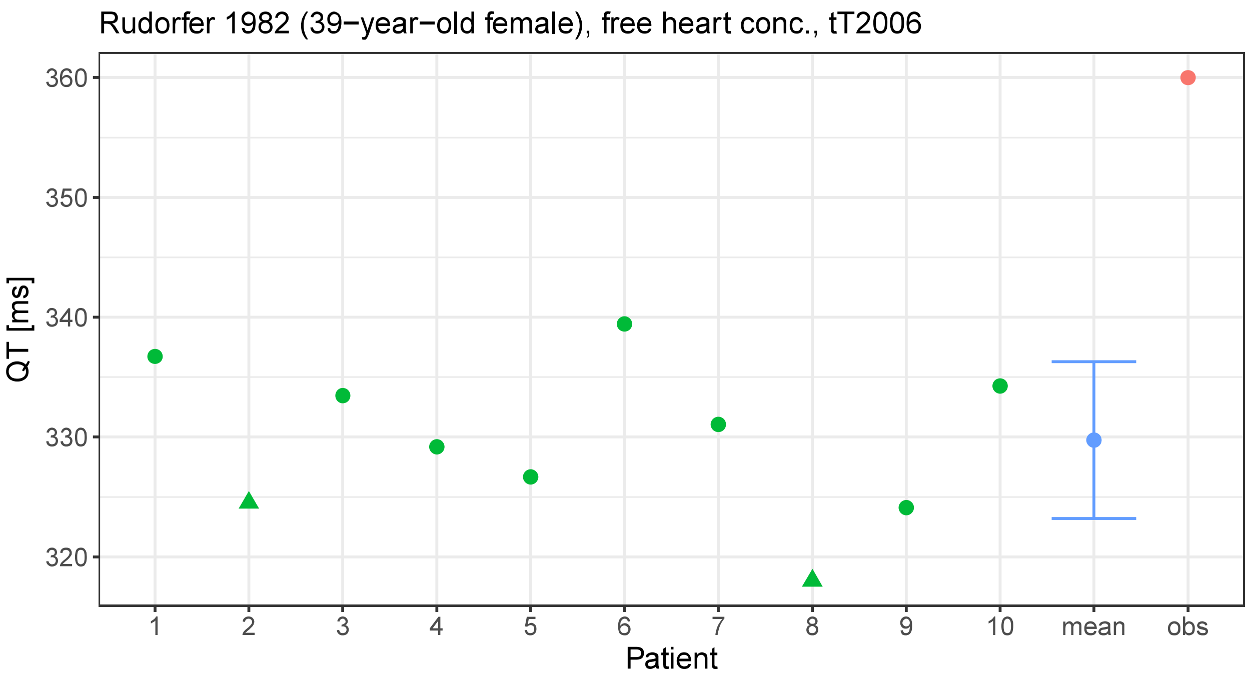

H

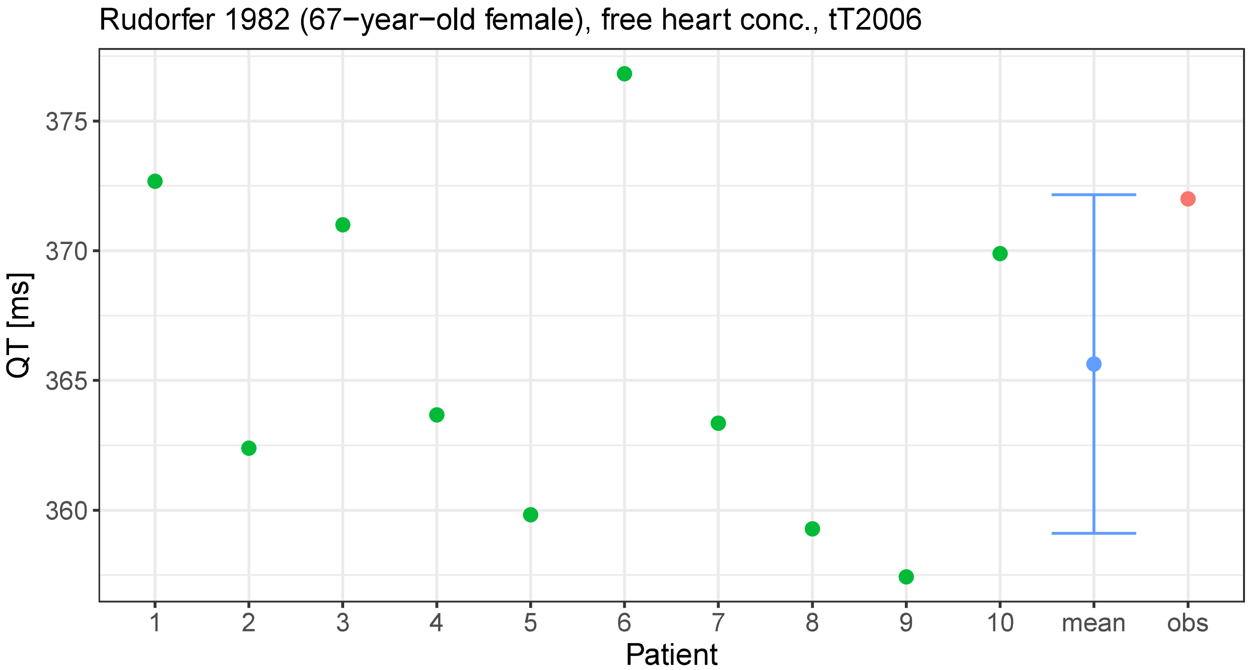

I

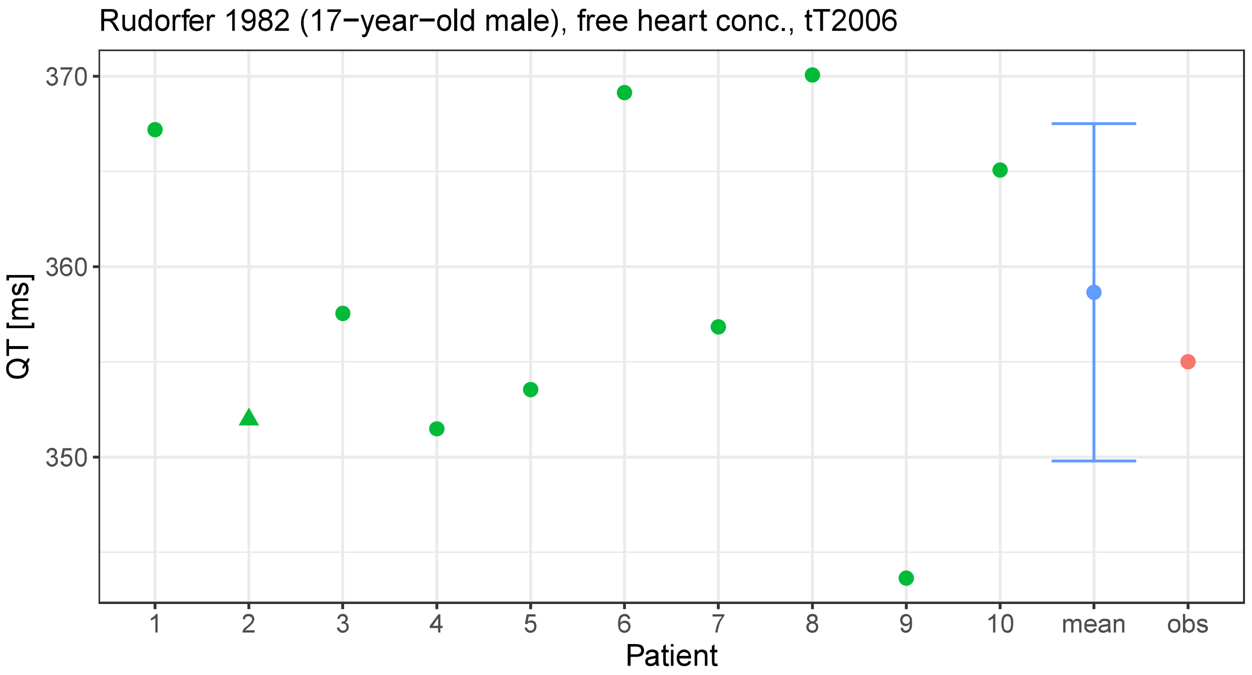

Figure 3 - Continued

J

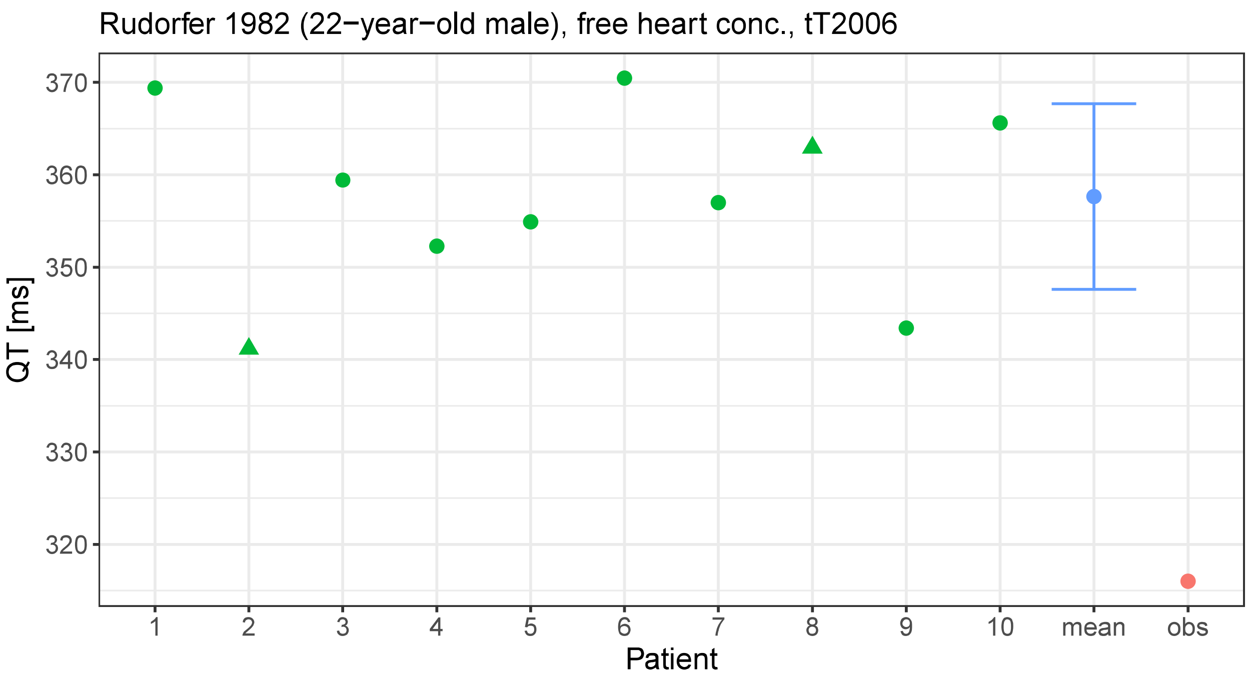

K

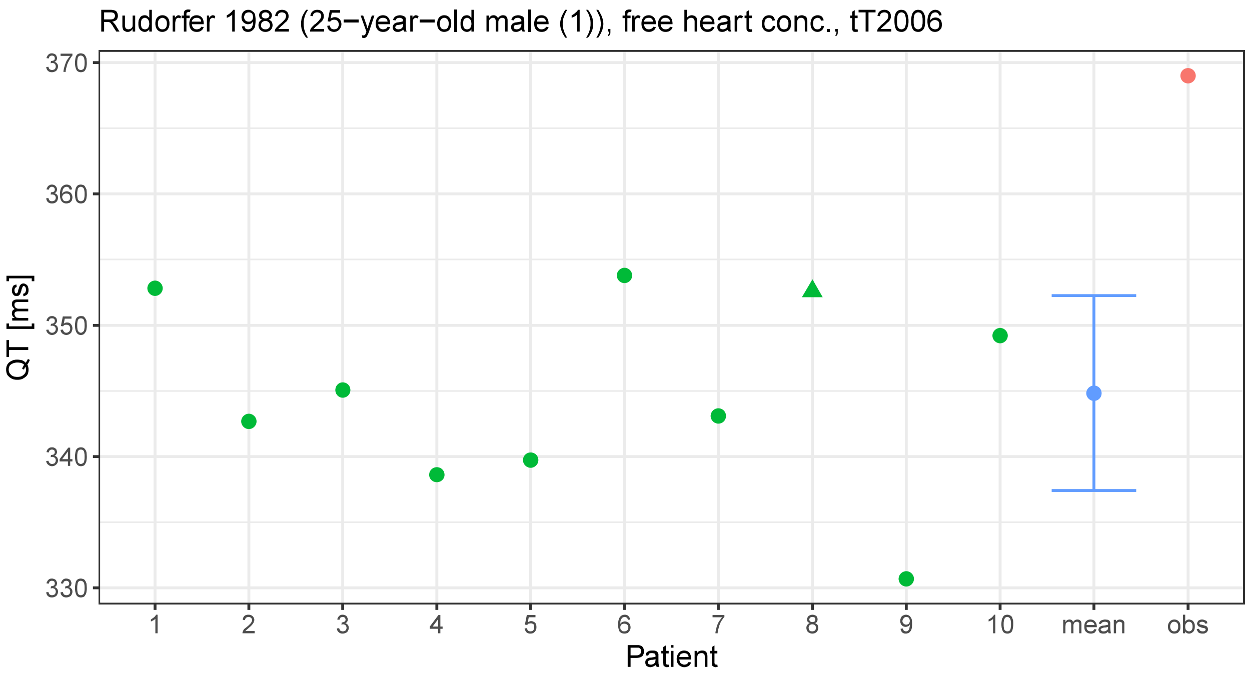

L

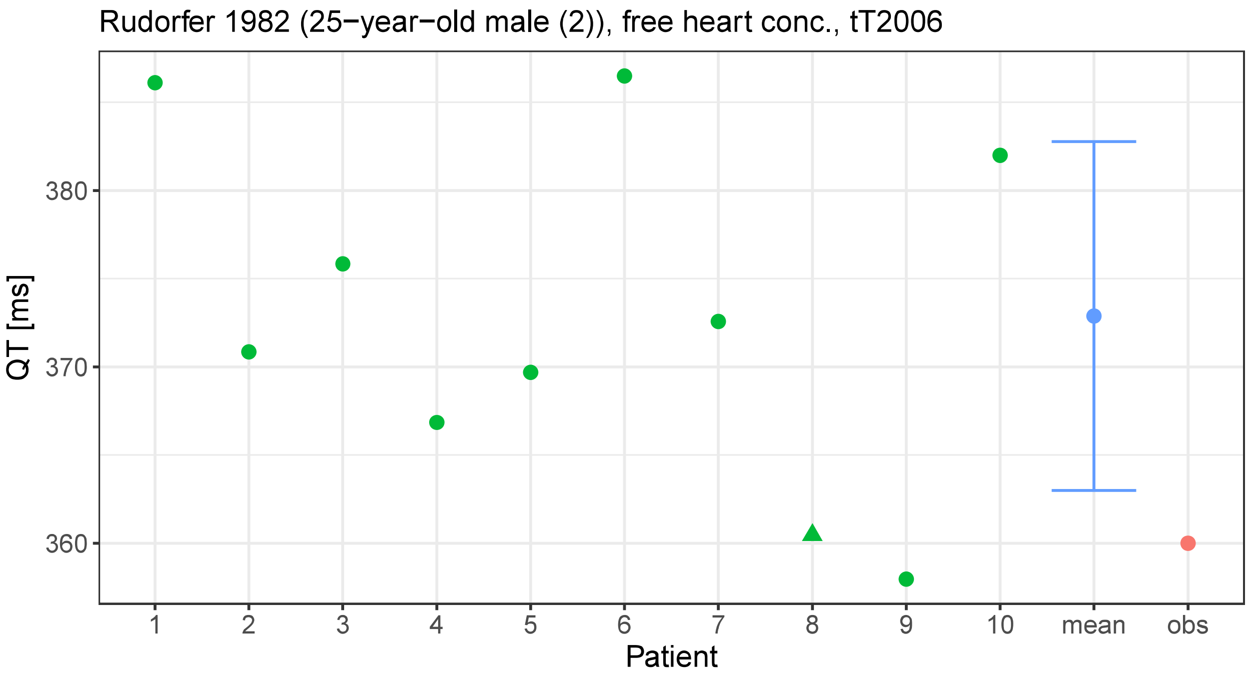

Figure 3 - Continued

M

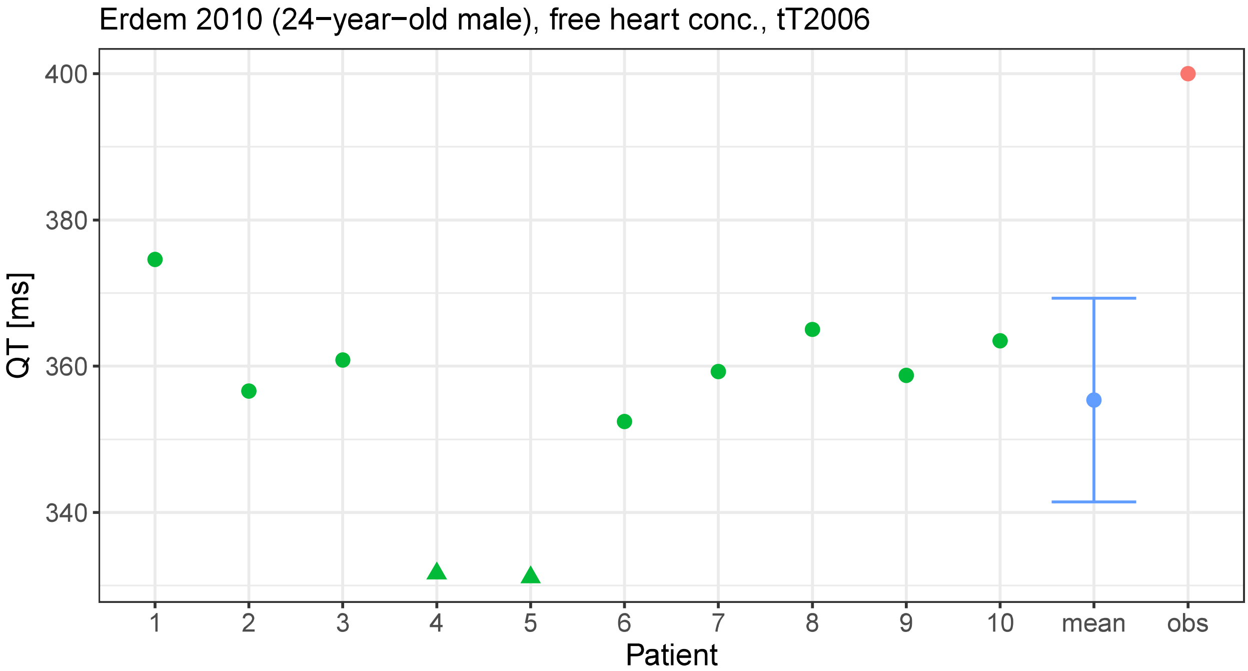

N

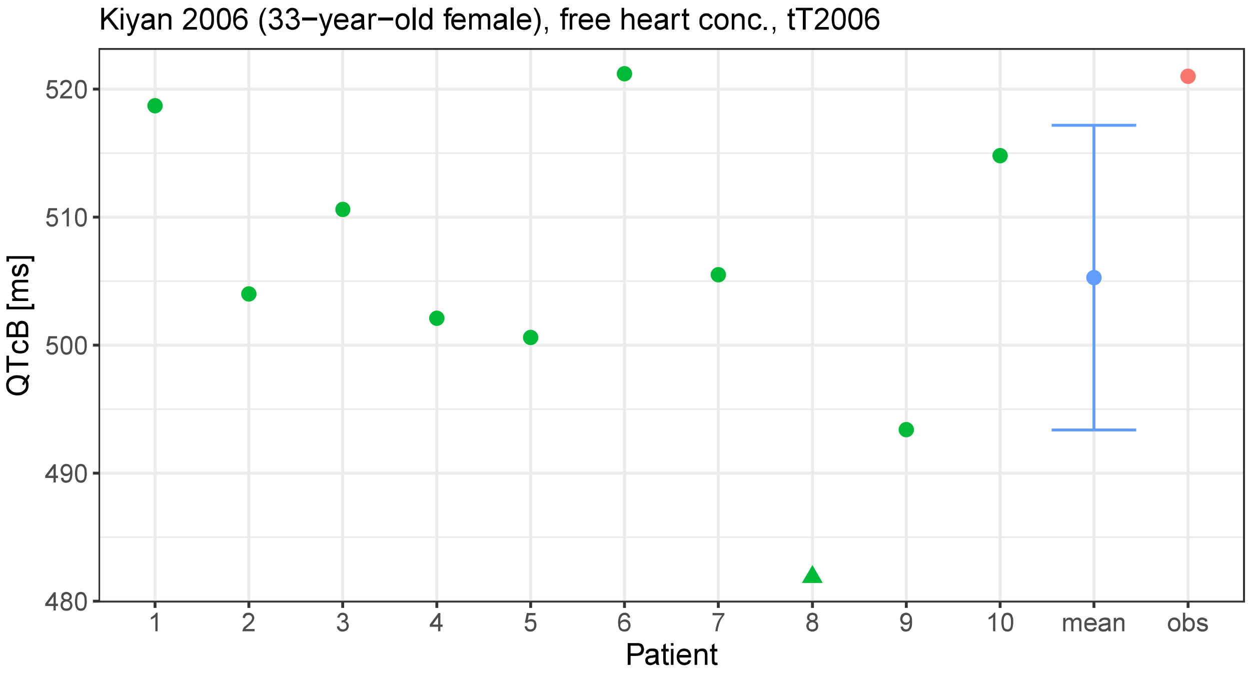

O

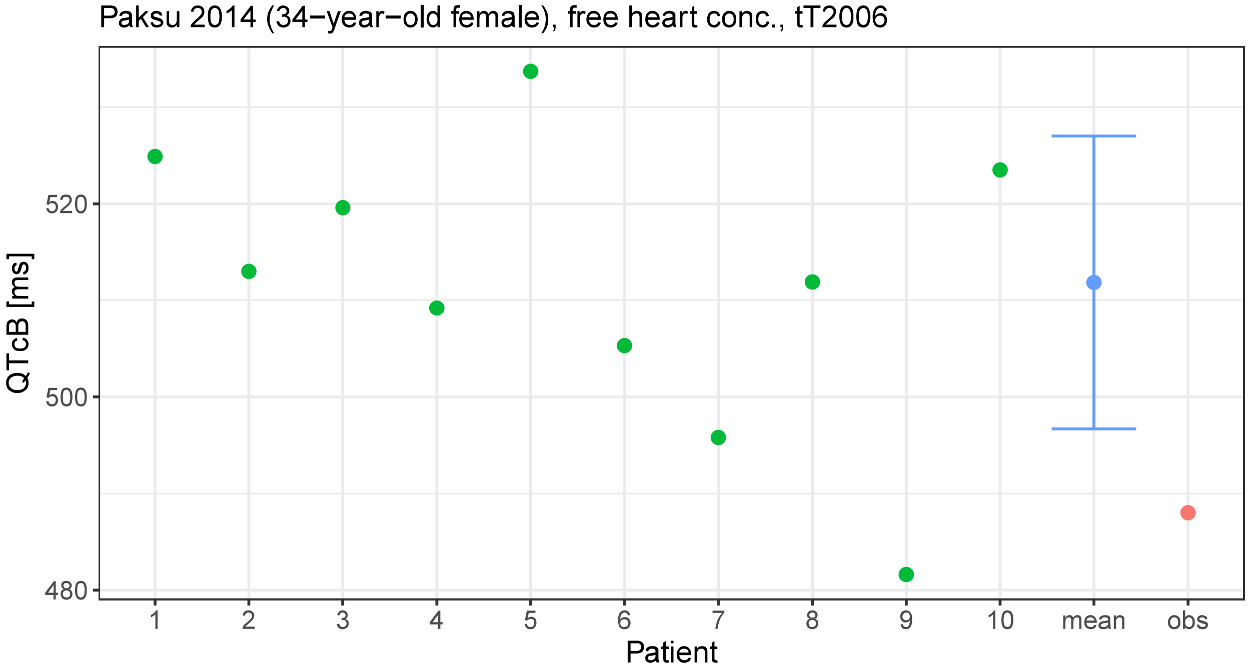

Figure 3 - Continued

P

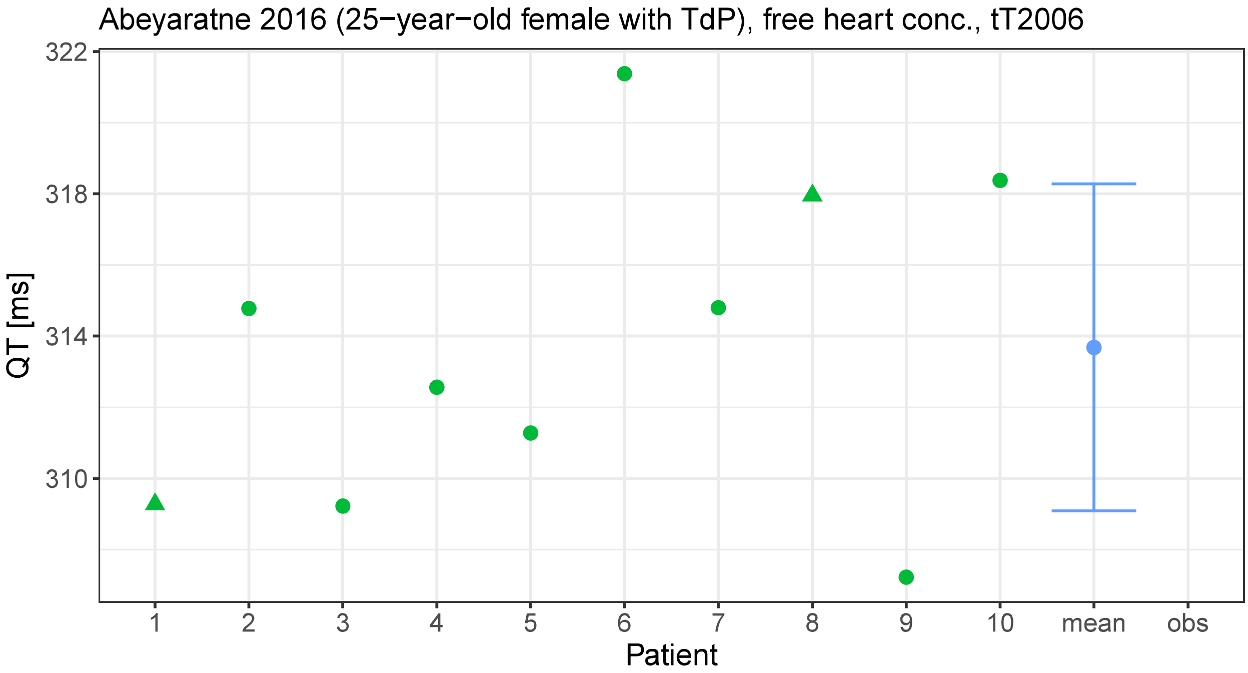

Q

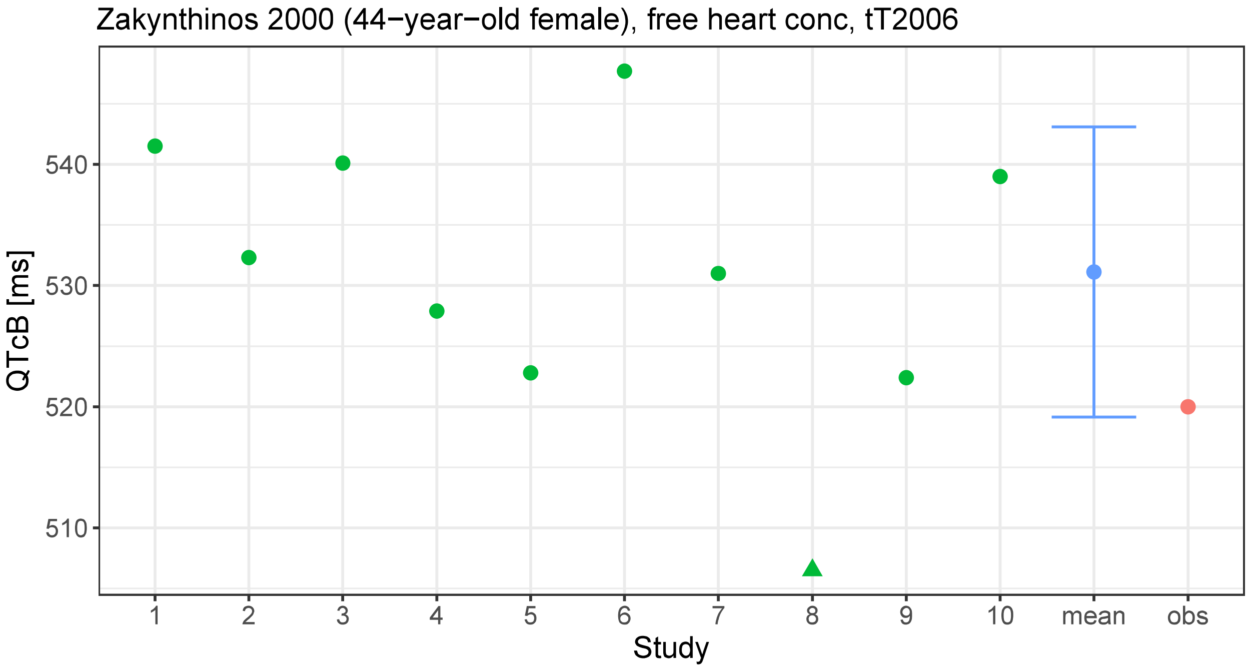

R

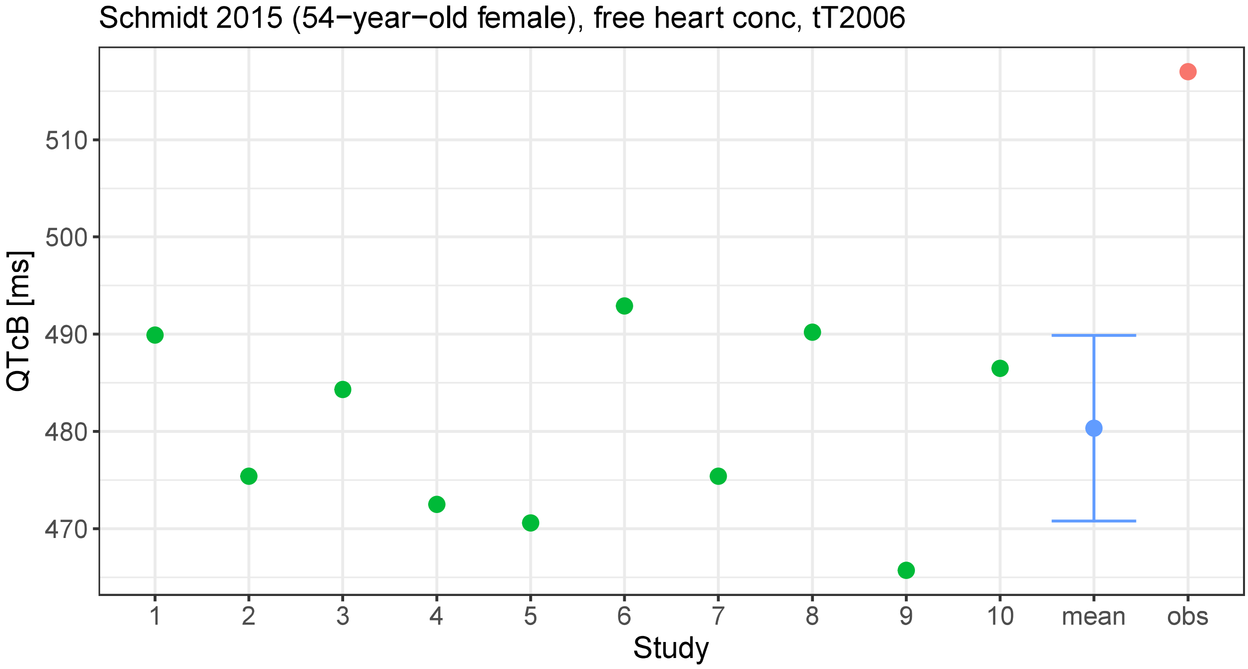

Supplement: Supplementary file 1 — Supplementary material 1 (PDF 6007 kb) [file 10928_2018_9597_MOESM1_ESM.pdf]
